# Near-duplicate contamination inflates brain MRI benchmark performance: a size-matched, multi-seed MedNeXt-B study

**DOI:** 10.3389/fmed.2026.1955863

**Published:** 2026-10-08

**Authors:** Yuhan Huang, Zhipeng Sun, Jinghui Chen, Tao Yang, Lianxin Xie, Xinchen Lin

**Affiliations:** 1 The First Clinical Medical College, The Affiliated People’s Hospital of Fujian University of Traditional Chinese Medicine, Fuzhou, Fujian, China; 2 Department of Interventional Radiology, The Affiliated People’s Hospital of Fujian University of Traditional Chinese Medicine, Fuzhou, Fujian, China

**Keywords:** brain tumor MRI, data leakage, medical image classification, medical image segmentation, MedNeXt, multi-task learning, near-duplicate detection

## Abstract

**Background:**

Pre-partitioned brain tumor MRI collections may place exact or near-duplicate slices on both sides of a benchmark split, but the magnitude and uncertainty of the resulting performance difference are rarely measured.

**Methods:**

A unified MedNeXt-B 2D model produced a four-class label and a tumor mask from a shared encoder. For each near-duplicate-screened fold, we constructed a replacement-based contaminated condition of identical size and tumor-class-by-plane composition; this condition was not the original unscreened training pool. Validation folds and the frozen 995-slice test set were identical. Constituent source-dataset matching was not possible because BRISC2025 provides no per-image source provenance. Both conditions were trained with seeds 42, 1,337, 2,024, 3,407, and 5,090. Primary uncertainty summaries used a 10,000-resample hierarchical bootstrap over seeds and inferred pseudo-patient clusters.

**Results:**

Across five seeds, screened accuracy was 0.9652 ± 0.0023 and mean class Dice was 0.7894 ± 0.0031 (mean ± sample SD). Accuracy was higher in the replacement-based contaminated condition by a mean difference of 0.0261 ± 0.0031 (range 0.0211–0.0281; hierarchical 95% CI, 0.0207–0.0318); corresponding mean differences were 0.0315 ± 0.0049 for mean class Dice (range, 0.0252–0.0375; hierarchical 95% CI, 0.0238–0.0394) and 0.0746 ± 0.0055 for meningioma recall (range, 0.0660–0.0792; hierarchical 95% CI, 0.0590–0.0911). The screening surrogate recovers only 5.8% of known same-patient slice pairs in an independent calibration dataset, so the screened condition may retain undetected related slices, and the intervals do not establish patient-level independence.

**Conclusion:**

Retaining detected test-linked near-duplicates was associated with higher slice-level benchmark performance in this replacement-matched exploratory analysis.

## 1 Introduction

Brain tumors cause high morbidity and mortality, impair cognition and neurological function, and remain a central concern of neuro-oncology (1, 2). Glioma, meningioma, and pituitary adenoma are the three common primary brain tumor entities encountered in clinical practice, and they differ in origin, biological behavior, imaging appearance, and management. Glioma arises from glial cells, grows infiltratively with irregular borders, and spans a wide malignancy range; it is the most common malignant primary brain tumor, and high-grade disease carries a poor prognosis, complicating surgery and radiotherapy (3). Meningioma arises from arachnoid cap cells, is usually extra-axial, and is the most common primary intracranial tumor; most cases are benign, but proximity to vessels and nerves makes accurate preoperative localization and volume assessment important (4). Pituitary adenoma sits in the sellar region and is often small, yet it can cause marked symptoms through hormone secretion or compression of the optic chiasm. The 2021 WHO classification of central nervous system tumors combines histology with molecular features and underlines how much accurate typing matters for treatment and prognosis (1).

Magnetic resonance imaging (MRI) provides excellent soft-tissue contrast and is the primary modality for detecting, localizing, classifying, grading, planning, and monitoring brain tumors (5). T1-weighted, contrast-enhanced T1-weighted (T1c), T2-weighted, and FLAIR sequences together characterize the tumor and its peritumoral environment. In routine clinical practice, however, MRI volume is large, and tumor appearance is heterogeneous. Reading slice-by-slice takes time and depends on reader experience and fatigue, introducing interobserver variation and the risk of missing small or ill-defined lesions. Automated analysis that is accurate, fast, and reproducible therefore has practical value for diagnostic throughput and precision oncology care.

Automated analysis serves two primary objectives: segmentation and classification. Segmentation defines tumor extent, volume, and treatment targets; classification supports diagnostic triage and subsequent clinical decision-making. Conventional pipelines optimize the two tasks separately, for example, U-Net or the self-configuring nnU-Net is deployed for segmentation (6, 7), and ResNet, ConvNeXt, or Swin Transformer for classification (8–10). Specialist designs of this kind often lead on a single metric, but deployment then needs several models, several inference pipelines, and separate calibration and monitoring for each. On imaging workstations, bedside devices, and resource-limited settings, repeated encoder passes, stacked memory peaks, and serial latency carry a direct computational cost. A shared encoder that returns a mask and a class probability in one forward pass lowers that cost and keeps both error analyses within one representation. However, such joint architectures may also compromise single-task accuracy, and joint optimization can transfer negatively, especially for blurred borders, tiny lesions, and imbalanced classes. Conversely, multi-task learning theory and recent surveys hold that jointly optimizing related tasks can act as mutual regularization and help a model learn transferable shared features when data are limited (11, 12).

MedNeXt adapts the ConvNeXt-style convolutional design to medical image segmentation through large-kernel depthwise convolutions, inverted bottlenecks, and a scalable hierarchical encoder–decoder (9, 13). We wrapped the published MedNeXt-B architecture as a shared feature extractor and added a lightweight classification head at the bottleneck. Data handling, augmentation, optimization, ensembling, and evaluation are specific to this study, so the model constitutes a local 2D multi-task implementation rather than the official training protocol. The 2D formulation is appropriate here because BRISC2025 is distributed as independent 2D images, and slice-wise inference reduces memory overhead and computational demand. However, it does not reproduce routine clinical interpretation, in which radiologists integrate complete volumes, multiple planes and multiple MRI sequences, and it lacks through-plane context. The resulting outputs should therefore be interpreted as benchmark-level slice predictions rather than volumetric clinical assessments.

Whether these comparisons are interpretable depends on how the data were split. BRISC2025 is distributed pre-partitioned and carries no patient identifiers. Slice-wise partitioning and test-linked near-duplicates can inflate medical-imaging performance estimates, while reporting frameworks emphasize transparent provenance, participant-level separation where possible, and the quantification of uncertainty (14–20). Counting near-duplicates alone is insufficient: in a converted-2D cohort split strictly by patient, 60.0% of test slices still had a near-duplicate neighbor at the selected threshold; thus, interpreting the raw rate requires a dataset-specific base rate (Supplementary Table S3).

Data leakage in medical-imaging benchmarks is not a single phenomenon, and separating its forms is central to this study. We distinguish five forms of train-test dependence. Exact duplication places byte-identical or pixel-identical images on both sides of a split, and cryptographic hashing removes it deterministically. Near-duplicate contamination places visually near-identical but non-identical images across the split. These images may be rescaled, recompressed, reformatted, or lightly reprocessed copies of the same acquisition; they survive exact-hash removal and require perceptual screening. Patient-level overlap places different images from the same patient or examination on both sides, enabling a model to exploit patient-specific anatomy even when no single image is duplicated. Slice-level dependence arises when anatomically adjacent slices in a volume lie on different sides, such that neighboring slices leak context. Conventional train-test leakage additionally covers preprocessing, feature selection, or normalization statistics estimated from the entire dataset before partitioning. These forms co-occur and are only partly detectable. Exact and near duplication are image-level phenomena and can be screened without identifiers, whereas patient-level and slice-level dependence require patient, examination, or slice-index metadata that pre-partitioned public collections such as BRISC2025 do not release. Prior studies show that leakage inflates reported accuracy in 2D brain-MRI classifiers and other medical-imaging pipelines (18–20). Those studies mostly document that leakage is present or estimate its aggregate effect. They rarely isolate near-duplicate contamination under a size- and composition-matched design or place its endpoint-specific estimates alongside model-modification results while preserving the different contrasts and estimands. Our objective is to measure, on a frozen test set and with a fixed unified architecture, the change in slice-level benchmark performance associated with retaining detected test-linked near-duplicates. We report this contrast separately from single-factor architectural and task-sharing modifications of the same model. The primary hypothesis, stated for this exploratory analysis, is that retaining detected test-linked near-duplicates in an otherwise size-matched and tumor-class-by-plane-matched training set is associated with higher slice-level test performance than screening them out.

We therefore estimated the benchmark contrast using a size-matched, multi-seed design. For every screened fold, a replacement-based contaminated training fold contained the same number of images and identical tumor-class-by-imaging-plane counts, while the validation fold and frozen test set remained unchanged. Within each stratum, screen-negative training images were replaced with detected test-linked near-duplicates. This constructed sensitivity condition differs from the original 4,955-image unscreened training pool and should not be interpreted as a reconstruction of that pool. The comparison incorporated five independent training seeds. Because per-image constituent source provenance was absent from the released metadata, source-dataset matching could not be implemented and remains a limitation.

The remaining experiments provide context rather than a formal ranking. We evaluated the unified model against nnU-Net v2 and three ImageNet-pretrained classifiers on the screened split, compared joint training with matched single-task controls, and tested six single-factor changes to the network and loss function (21–26). Because these analyses use different endpoints and experimental contrasts, their point estimates are described separately; we make no claim that one category of effect is definitively larger than another.

We also report uncertainty intervals for selected descriptive endpoints, sensitivity analyses over inferred pseudo-patient groups, calibration, empty-mask rates, device-matched compute metrics, a fully specified Grad-CAM audit of the shared bottleneck, and exploratory zero-shot and few-shot transfer to public converted-slice cohorts. These analyses remain slice-level and exploratory because reliable patient identifiers are unavailable.

The primary result is a size-matched, multi-seed comparison of near-duplicate-screened folds and a replacement-based contaminated condition on a fixed test set. Supporting analyses characterize a near-duplicate-screened 2D MedNeXt-B model, compare joint versus single-task training, and evaluate six exploratory single-factor interventions selected for this analysis. We make no claim of patient-level leakage elimination, causal retrieval behavior, clinical external validation, or universal architectural superiority.

To make the scope explicit, we separate the study's methodological contributions from implementation choices and exploratory context, and each stated contribution has a corresponding method and a directly reported result. The methodological contributions are fourfold. First, a size-matched, tumor-class-by-plane-matched, multi-seed design isolates near-duplicate contamination from training-set size and measured composition. Second, we calibrate the perceptual-hash near-duplicate screen against an independent identifier-carrying dataset and report its precision and recall, rendering its detection limits explicit. Third, a hierarchical seed-plus-pseudo-cluster bootstrap propagates training-seed and within-test clustering uncertainty into paired contamination-effect intervals. Fourth, we report endpoint-specific contamination estimates and the separate architectural and task-sharing results on the same frozen test set, while retaining their distinct endpoints, contrasts, estimands and uncertainty procedures. These contextual comparisons are not treated as commensurate effects or ranked inferentially. The 2D MedNeXt-B backbone, the joint segmentation-classification head, the augmentation protocol, and the ensembling scheme are implementation choices, not claimed novelties. The cross-dataset transfer, gradient-weighted class activation mapping (Grad-CAM) attribution, calibration, few-shot and single-factor intervention analyses are exploratory context. Figure 1 summarizes the study design and separates the data-cleaning, model-development, and evaluation processes.

**Figure 1:**
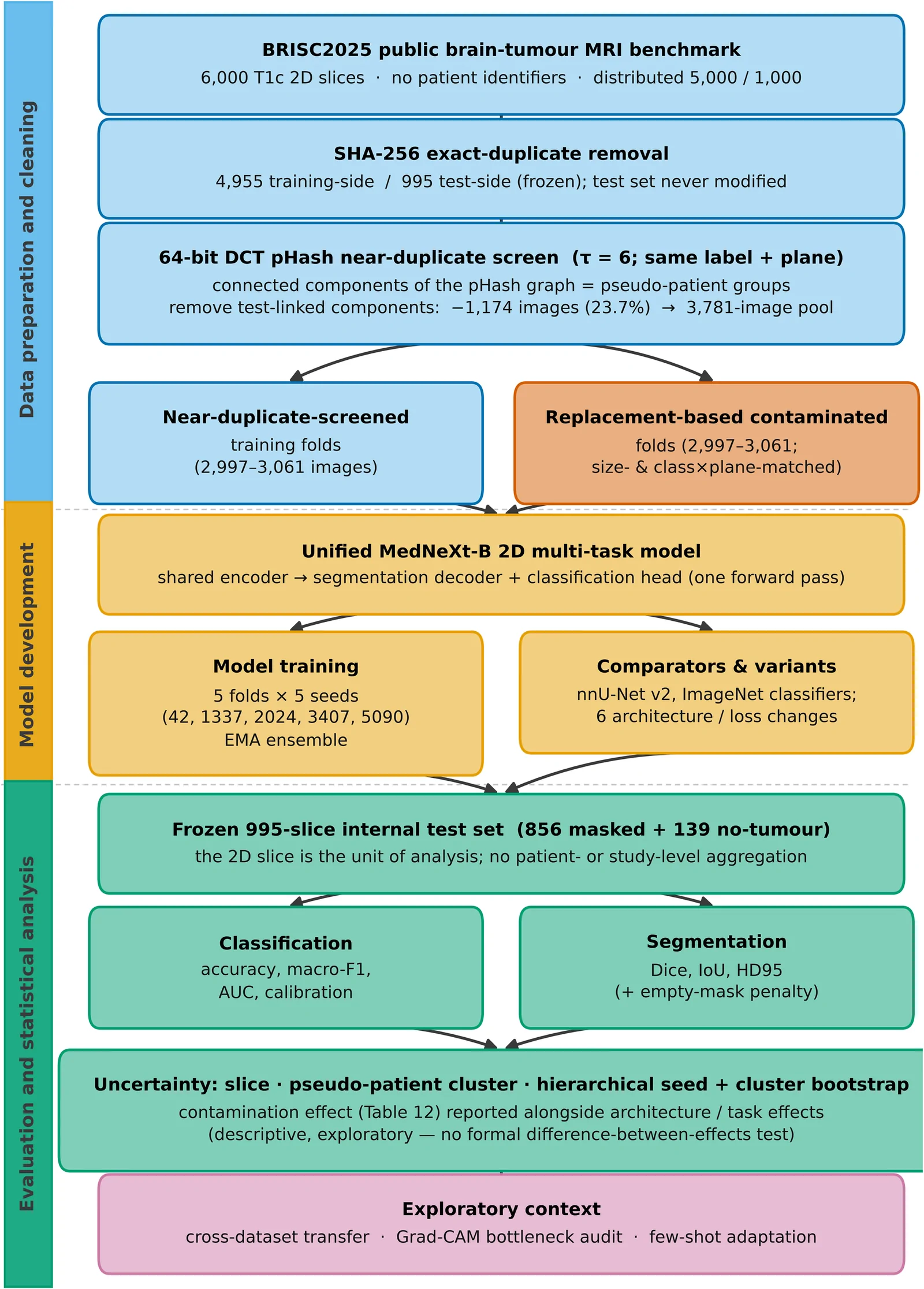
Study design and analysis workflow.

## 2 Materials and methods

### 2.1 Dataset

The primary data derive from the public BRISC2025 brain tumor MRI dataset (27). Its source publication describes 6,000 contrast-enhanced T1-weighted brain MRI images collated from several public sources and annotated by certified radiologists and physicians, covering no tumor, glioma, meningioma, and pituitary across the axial, coronal, and sagittal planes. The task is four-class classification with tumor segmentation on a single slice. BRISC2025 is distributed as 6,000 slices in a 5,000/1,000 training/test partition, with 512 × 512 JPEG images and binary tumor masks for the subset that carries segmentation labels. Before training, byte-identical images were removed by SHA-256 hashing, leaving 4,955 training-set and 995 test-set images; the test set was then frozen. Near-duplicate screening left 3,781 images for fivefold training and validation. The release used in this study did not include a per-image field identifying the constituent public source dataset; consequently, source-level stratification and source-matched replacement could not be performed.

### 2.2 Data partitioning and the unit of analysis

BRISC2025 is distributed as independent 2D slices. It releases no patient, examination, series, acquisition, or volume identifier and no slice-index field. The observable unit of modeling and evaluation is therefore the individual 2D image (slice). Each file is treated as one example, and no image was aggregated to a patient-level or study-level prediction. Because the released data contain no verified patient field, we could not establish patient-exclusive training, validation, or test partitions before slice extraction; the distributed 5,000/1,000 partition is defined at the image level. For the same reason, we cannot determine whether all slices of a given patient and examination fall in one partition, whether multiple examinations from one patient appear in different partitions, or whether anatomically adjacent slices from one volume were separated across partitions. The near-duplicate screen detects only image pairs whose appearance is near-identical. It flags some near-identical adjacent slices but does not recover full patient-level or volume-level grouping. We therefore formed pHash-derived pseudo-patient groups as an explicitly imperfect surrogate. These groups were used to remove detected test-linked components, to keep detected groups intact when constructing the five development folds, and as resampling units for cluster-based uncertainty. They were never interpreted as verified patient identities and were not used to tune the architecture, hyperparameters, or model selection criteria. Under this surrogate, training-to-validation pseudo-patient overlap was zero, and no detected test-linked group entered the screened training folds. However, the surrogate recovers only 5.8% of known same-patient slice pairs in an independent calibration dataset, so undetected same-patient or same-examination slices may still straddle the partitions. For the units that are observable, the distributed split contained 5,000 training-side and 1,000 test-side images; SHA-256 exact-duplicate removal reduced these to 4,955 and 995; near-duplicate screening left a 3,781-image pool for grouped fivefold training and validation; and the frozen internal test set comprised 995 images, of which 856 carried segmentation masks, and 139 were no-tumor. True patient and examination counts are unavailable. All reported point estimates use the 2D slice as the statistical unit. We computed confidence intervals using slice bootstrap and pseudo-patient-cluster bootstrap, and the primary contamination intervals using a hierarchical seed-plus-pseudo-cluster bootstrap; none of these are patient-level intervals. Because the partition cannot be shown to be patient-independent, every performance value is a slice-level benchmark estimate, and we confine all patient-level and clinical interpretations to statements this design can support.

### 2.3 Unified model

The model takes a 256 × 256 single-channel slice and uses MedNeXt-B as a shared encoder. At a base width of 32, the encoder produces feature maps at 256 × 256, 128 × 128, 64 × 64, and 32 × 32 and a 512-channel 16 × 16 bottleneck. Although BRISC supplies binary tumor masks, the segmentation target has four semantic labels. Background pixels are label 0; every foreground pixel in a masked tumor image receives that image's class label (glioma 1, meningioma 2, or pituitary 3). No-tumor images and images without a supplied mask receive an all-zero placeholder tensor but are marked has_mask=false, so they contribute to classification loss only and never to segmentation loss or segmentation metrics. Segmentation evaluation is restricted to the 856 images with supplied masks. The native decoder outputs four logits, and the objective combines equal-weight Dice and segmentation cross-entropy on masked slices with classification cross-entropy using a label smoothing factor of 0.05 on all slices. The classification head applies global average pooling, LayerNorm, a 512-to-256 linear layer, Gaussian error linear unit (GELU), dropout at 0.3, and a four-class output layer (Figure 2).

**Figure 2:**
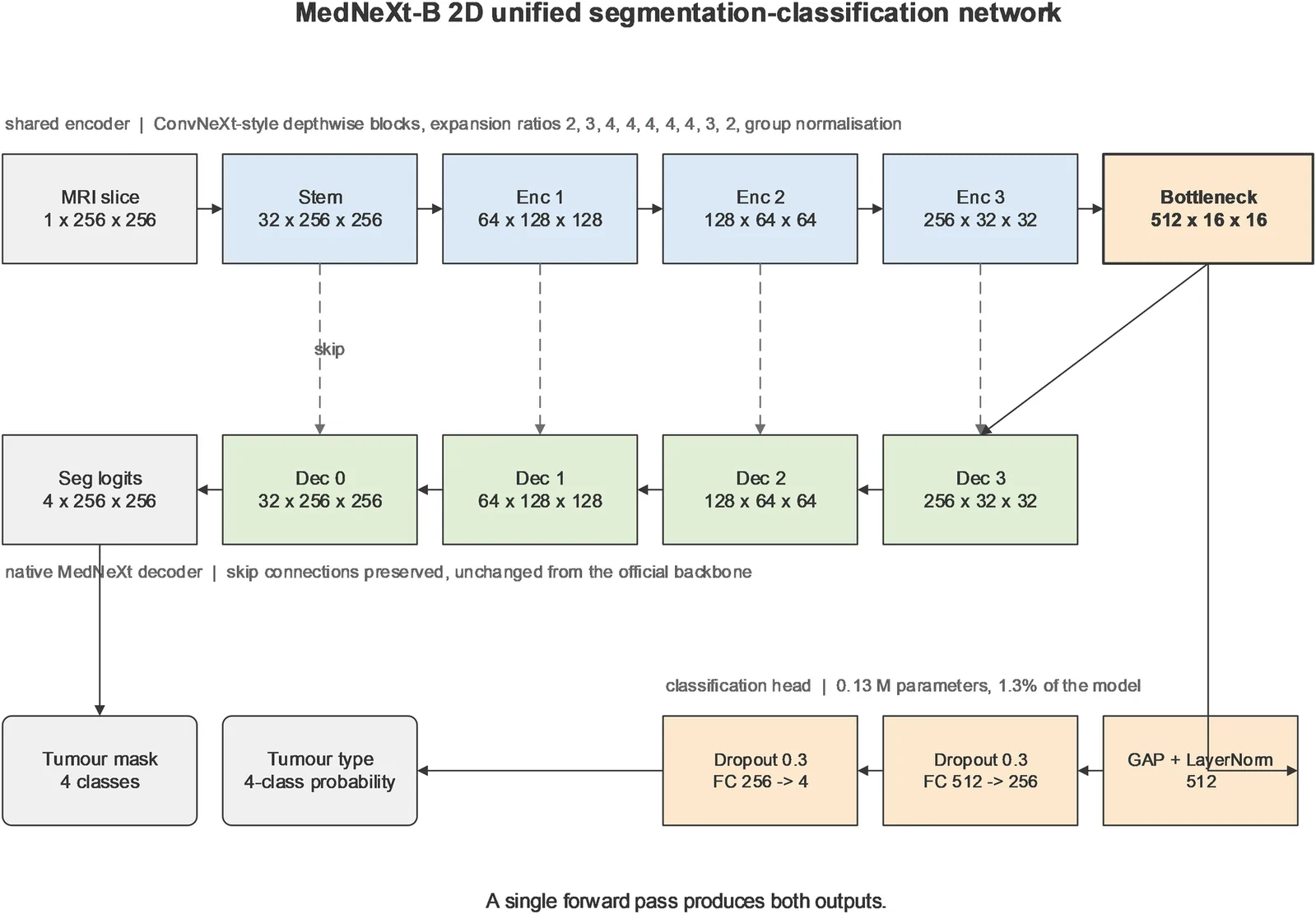
MedNeXt-B 2D multi-task architecture. A shared encoder feeds the native segmentation decoder and a bottleneck classification head in one forward pass.

Sharing the encoder eliminates the need for a dedicated second architecture; the classification head reuses the segmentation encoder instead of invoking a separate classifier. This design reduces the overall model count and parameter storage. However, it carries no guarantee regarding task accuracy, calibration, latency, or generalization; consequently, we measured each of these endpoints separately.

### 2.4 Algorithmic interventions

Every intervention follows a single-factor rule: each change touches one module, one loss term, or one supervision point, while the backbone, input size, number of epochs, optimizer, learning-rate schedule, and split remain identical. The variants are Attention Gate, ASPP, MS-FE, high-resolution multi-scale feature enhancement (HR-MSFE), Focal Tversky, and Deep Supervision (21–26). Their insertion sites and endpoints selected for this exploratory analysis are reported in Supplementary Table S2. No dated protocol or preregistration was available; therefore, the intervention and multiplicity analyses are explicitly *post hoc* and exploratory.

### 2.5 Training and statistics

We trained MedNeXt-B 2D for 100 epochs with a batch size of 16 and AdamW (initial learning rate 3×10^−4^, weight decay 5×10^−2^) (28), using a 5-epoch linear warmup, cosine decay, gradient clipping at 1.0, exponential moving average (EMA) decay of 0.999, and early stopping. Folds were grouped by the inferred pseudo-patient identifier and stratified by tumor label. Training augmentation was synchronized between images and masks and included horizontal and vertical flips, 90° and ± 20° rotations, brightness/contrast jitter, blur, noise, elastic deformation, grid distortion and coarse dropout. These transformations were used as label-preserving robustness perturbations rather than as simulations of clinically acquired anatomy. Vertical flips, large rotations and spatial deformations may create anatomically atypical appearances, which limits their clinical interpretation. Validation and testing used no augmentation or test-time augmentation. Each reported model is the probability-space ensemble of five fold-level EMA checkpoints.

### 2.6 Experimental configuration

MedNeXt-B 2D and its six variants used 256 × 256 inputs, batch size 16, five folds, and mixed precision. The resolution-matched retest used 512 × 512 inputs and a batch size of 4. nnU-Net v2 used its standard self-configuring 2D training procedure and stands as a strong specialist rather than a budget-matched control. Both arms used the same recorded interpreter and configuration. For this rerun, Python, NumPy, PyTorch, and Compute Unified Device Architecture (CUDA) random generators were seeded with the base seed plus fold. Fixed seeds, archived checkpoints, and recorded hashes support traceability. Because deterministic algorithms were requested with warn-only behavior, operations without a deterministic CUDA implementation fall back to non-deterministic kernels, so bit-identical reproduction across executions is not guaranteed. The seed-42 screened ensemble described in Tables 1–3 and in the task-sharing and calibration analyses is therefore the earlier frozen run (accuracy 0.9648) and is not identical to the seed-42 run of the five-seed rerun (accuracy 0.9638); per-case output arrays, fold checkpoints and file hashes for both runs are deposited.

**Table 1:** Internal-test performance of the unified model.

Metric	Value	95% CI/note
Accuracy	0.9648	0.9528–0.9759
Macro-F1	0.9614	0.9469–0.9738
Macro-AUC	0.9984	0.9974–0.9992
Brier score	0.0540	0.0387–0.0706; lower is better
ECE (15-bin, top-label)	0.0324	lower is better
Mean class Dice (pooled)	0.7941	0.7743–0.8130; class macro 0.7899 (0.7703–0.8088); per-case foreground 0.8127 (0.7985–0.8267); the three estimators share one 5,000-resample case bootstrap
Glioma Dice	0.7162	0.6832–0.7484
Meningioma Dice	0.8502	0.8238–0.8736
Pituitary Dice	0.8035	0.7810–0.8232
WT Dice	0.8290	0.8176–0.8407
WT HD95 (pixels)	8.343	7.472–9.363; *n*=850 finite. Sensitivity with six empty predictions assigned the 256 × 256 image diagonal (362.04 px): mean 10.823, 95% slice-bootstrap CI 8.807–13.107.

**Table 2:** Internal-test confusion matrix (rows true, columns predicted).

True/Pred	No-tumor	Glioma	Meningioma	Pituitary
No tumor	139	0	0	0
Glioma	0	249	3	1
Meningioma	20	4	274	5
Pituitary	0	0	2	298

**Table 3:** Per-class sensitivity and specificity on the internal test set.

Class	Sensitivity (95% Wilson CI)	Specificity (95% Wilson CI)
No tumor	1.000 (0.973–1.000)	0.977 (0.964–0.985)
Glioma	0.984 (0.960–0.994)	0.995 (0.986–0.998)
Meningioma	0.904 (0.866–0.933)	0.993 (0.983–0.997)
Pituitary	0.993 (0.976–0.998)	0.991 (0.981–0.996)

The two-task objective was $L=\lambda_{\mathrm{seg}}\left(0.5L_{\mathrm{Dice}}+0.5L_{\mathrm{CE},\;\mathrm{seg}}\right)\;+$ $\lambda_{\mathrm{cls}}L_{\mathrm{CE},\;\mathrm{cls}},\;\mathrm{with}\;\;\lambda_{\mathrm{seg}}=\lambda_{\mathrm{cls}}=1.$

Classification outcomes were accuracy, macro-F1, weighted-F1, macro-AUC, confusion matrices, expected calibration error (ECE), maximum calibration error, and Brier score (29). Segmentation outcomes were Dice, intersection over union, and HD95 (16). The nonempty-union and true-class-only estimands were selected for this exploratory analysis rather than prospectively specified. For the contamination comparison, five principal descriptive endpoints were reported: accuracy, mean class Dice, whole-tumor Dice, whole-tumor HD95, and meningioma recall. For each endpoint, the five seed-specific contaminated-minus-screened differences, their mean, sample SD, and range were calculated. A Student-*t* interval over the five seed differences (df = 4) is shown only as an indicative small-sample summary and is not the primary interval. The primary uncertainty summary is a 10,000-resample hierarchical percentile bootstrap (30) (random seed 20260828): each replicate samples five training seeds with replacement, samples inferred pseudo-patient clusters with replacement within each selected seed, retains all slices from every sampled cluster with multiplicity, recomputes both arms on the same sampled indices, and averages the paired differences across sampled seeds; the 2.5th and 97.5th percentiles define the interval. For HD95, the primary paired analysis uses only test slices with finite HD95 in both arms within a seed (*n* = 847, 850, 849, 849, and 851); a sensitivity analysis assigns an undefined HD95 the maximum 256 × 256 image diagonal (362.04 pixels) and includes all 856 masked slices. Because the grouping recovers only 5.8% of known same-patient pairs, neither the hierarchical interval nor slice-level McNemar, Wilcoxon, and DeLong (31) analyses establish patient-level inference. All hypothesis tests and multiplicity groupings are *post hoc* and exploratory. Descriptive intervals reported elsewhere are taken from the archived bootstrap outputs deposited with the analysis code: the three Table 1 mean-Dice estimators share one 5,000-resample case bootstrap (seed 42), the remaining Table 1 entries use a 1,000-resample slice bootstrap, and the paired calibration differences reported in Section 3.3 use a 2,000-resample paired case bootstrap (seed 42) in which both models are scored on identical resampled case indices.

### 2.7 Near-duplicate screening and the contamination experiment

We screened test-linked near-duplicates before training. A training image was flagged if an SHA-256 hash, an inferred duplicate group, an inferred pseudo-patient identifier, or a same-label and same-plane perceptual-hash neighbor linked it to the frozen test set. Pseudo-patient groups are connected components of a 64-bit pHash graph at a Hamming threshold of 6 and are not verified patient identities. Calibration on an independent dataset yielded 81.6% precision but only 5.8% recall for known same-patient slice pairs; the procedure is therefore a low-recall near-duplicate screen, not a true patient-level separation, and the screened condition may retain undetected related slices. For the experiment, each replacement-based contaminated training fold was identical in size to its screened counterpart (2,997–3,061 images), exactly matched on tumor class by imaging plane, and paired with the same validation fold. Within strata, a total of 1,135–1,151 screen-negative records were replaced with detected test-linked records per fold. This constructed condition is not the original unscreened 4,955-image training pool. Training/validation pseudo-patient overlap was zero only under the implemented detector. Per-image source provenance was unavailable; thus, the constituent source datasets could not be included in the matching variables. Both conditions were trained with seeds 42, 1,337, 2,024, 3,407, and 5,090 and evaluated on the same frozen 995-slice test set.

The near-duplicate screen operated in two deterministic stages. First, we removed exact duplicates by comparing SHA-256 digests of the stored image bytes, collapsing byte-identical files before learning or fold construction. Second, we computed pHash with ImageHash 4.3.1 (imagehash.phash) and Pillow 10.3.0. The implementation converted each image to 8-bit grayscale (L mode), resized it to 32 × 32 pixels using Pillow's Lanczos resampling, applied a two-dimensional discrete cosine transform, retained the upper-left 8 × 8 low-frequency block, and compared all 64 coefficients, including the direct-current coefficient, with the block median. Coefficients above the median produced one bits, and the remainder produced zero bits, yielding a 64-bit code (hash_size = 8, default highfreq_factor = 4). Thus, the hash describes perceptual image content rather than compressed file bytes and can match reformatted or lightly reprocessed copies that SHA-256 treats as distinct. Two images were linked when they shared the same class label and imaging plane and their codes differed by a Hamming distance of at most six bits (*τ* = 6). Links were merged transitively: images were nodes, qualifying links were edges, and connected components defined the groups. For screening, components were computed over the combined training and frozen-test sides, and every training image in a component containing a test image was removed. All 1,174 excluded images (23.7% of the 4,955-image pool) came from the training side; the 995-image test set was never reduced, and validation folds were drawn only from the screened training pool. The threshold was chosen from the calibration sweep in Supplementary Tables S3–S4, not from BRISC test outcomes, and then applied identically to both training conditions. Because no dated preregistration exists, we report *τ* as a fixed analysis choice rather than a prospectively specified criterion. At *τ* = 6, precision against known same-patient pairs was 0.816 and recall was 0.058. Relaxing *τ* to 8 or 10 raised recall to 0.097 and 0.140 but reduced precision to 0.614 and 0.391. Tightening *τ* to 2 or 4 yielded recalls of 0.007 and 0.025. We therefore selected *τ* = 6 to remove detectable test-linked near-duplicates while limiting exclusions of visually similar images. This image-level screen supplements but does not replace strict patient-level separation, which the dataset does not permit.

### 2.8 Pseudo-patient grouping

Pseudo-patient groups are defined by the same perceptual-hash graph used for screening; the two constructs are identical. A pseudo-patient group is a connected component of the *τ* = 6 pHash graph, where nodes are images and an edge joins two images that share the class label and imaging plane and whose 64-bit codes differ by at most six bits. Membership is inferred only from image content and the two categorical fields. It uses no filename, acquisition metadata, timestamp, model prediction, or manual annotation, so grouping is independent of model outputs and evaluated outcomes. Images with no qualifying neighbor form singleton groups of size one; no image is unassigned. Grouping was computed for the relevant pool. Components within the frozen test set defined bootstrap clusters, components over the combined training and test sets defined screening links, and components within the screened development pool supplied the grouping variable for StratifiedGroupKFold. The latter stratified folds by tumor label while keeping each detected pseudo-patient component wholly within either training or validation for a given fold. On the frozen 995-slice test set, the procedure yielded 872 groups (mean size 1.14, maximum 15, with 80.4% of slices in singleton groups); the 856 masked slices formed 747 groups, and no group spanned tumor classes. Group fidelity is characterized only indirectly through the calibration in Supplementary Tables S3–S4 (precision 0.816, recall 0.058 for known same-patient pairs). Pseudo-patient groups therefore served three purposes: defining the near-duplicate screen, constraining development-fold construction under the implemented detector, and supplying resampling units for cluster and hierarchical bootstraps. They were not verified patient identifiers and were not used to tune hyperparameters or select models. Algorithm 1 gives the stepwise procedure.

Algorithm 1 **Pseudo-patient grouping and near-duplicate screening.**

Input: training images Xtrain and frozen test images Xtest after SHA-256 exact-duplicate removal; class label c(x); imaging plane pl(x); Hamming threshold *τ* = 6.

Precondition: byte-identical images have been removed by SHA-256 hashing before pHash graph construction.

1. for each image x in Xtrain ∪ Xtest: h(x) <- imagehash.phash(x, hash_size=8, highfreq_factor=4) # 64-bit pHash
2. define BuildGraph(S): V <- S; E <- {(x,y): c(x)=c(y) AND pl(x)=pl(y) AND Hamming(h(x),h(y)) <= *τ*}; return G=(V,E)
3. G_all <- BuildGraph(Xtrain ∪ Xtest); C_all <- connected_components(G_all) # transitive links are merged
4. Xexcluded <- union over q in C_all with q ∩ Xtest ≠ ∅ of (q ∩ Xtrain); Xdev <- Xtrain \ Xexcluded # test images are never removed
5. G_dev <- BuildGraph(Xdev); P_dev <- connected_components(G_dev)
6. folds <- StratifiedGroupKFold(n_splits=5, y=c(x), groups=component_id(x; P_dev)); keep every detected component wholly within training or validation
7. G_test <- BuildGraph(Xtest); P_test <- connected_components(G_test)
8. cluster bootstrap: resample components in P_test with replacement and retain all member slices with multiplicity
9. hierarchical contamination bootstrap: resample training seeds with replacement; within each selected seed, resample P_test components with replacement; recompute paired contaminated-minus-screened differences on identical sampled test indices; average across sampled seeds

Output: screened development pool Xdev; excluded training images Xexcluded; development groups P_dev; test bootstrap groups P_test. All component labels are inferred pseudo-patient surrogates, not verified patient identities.

### 2.9 Exploratory cross-dataset transfer evaluation

Exploratory cross-dataset transfer evaluation used public converted 2D slices from the Medical Segmentation Decathlon glioma cohort (32), BraTS-MEN 2023 (33), and a public pituitary contrast-enhanced T1 dataset (34). These resources differ from BRISC in imaging sequence, preprocessing, and label definitions, and they were not assembled as an independent patient-level clinical validation cohort for this study. No independent no-tumor cohort passed the overlap screen; consequently, cross-dataset four-class specificity and AUC are not estimable. The analysis therefore describes technical transfer behavior only.

## 3 Results

### 3.1 Internal performance of the unified model

On the frozen internal test set, the previously frozen seed-42 ensemble reached an accuracy of 0.9648 (95% slice-bootstrap CI 0.9528–0.9759), a macro-F1 of 0.9614, a macro-AUC of 0.9984, a pooled mean class Dice of 0.7941 (0.7743–0.8130), and a whole-tumor Dice of 0.8290 (Figure 3). Across the five newly rerun screened five-fold ensembles, accuracy was 0.9652 ± 0.0023 and mean class Dice was 0.7894 ± 0.0031 (mean ± sample SD). Table 3 reports Wilson 95% confidence intervals for each class-specific sensitivity and specificity of the previously frozen ensemble. Its confusion matrix shows a notable error pattern: 20 meningioma slices were classified as no tumor (Table 2). These are slice-level benchmark results and not estimates of patient-level diagnostic performance.

**Figure 3:**
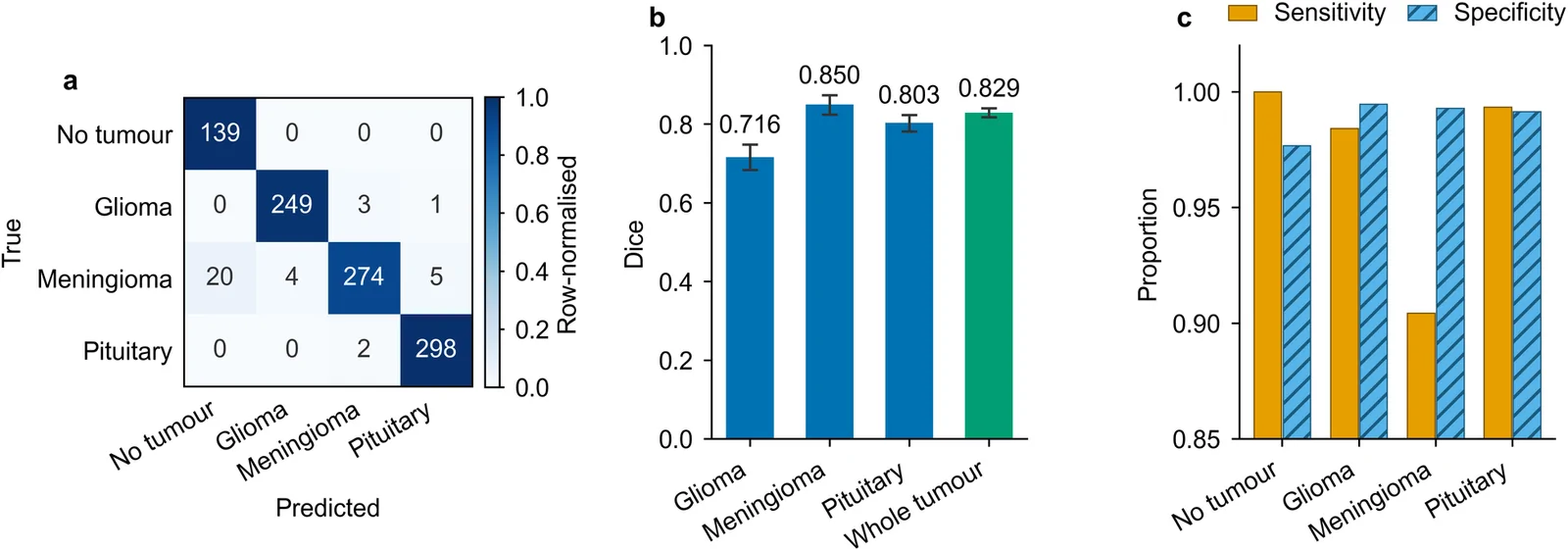
Internal-test performance. **(a)** Four-class confusion matrix. **(b)** Class-specific and whole-tumor Dice with bootstrap intervals. **(c)** Sensitivity and specificity.

A *post hoc* tumor-versus-no-tumor analysis yielded an AUC of 0.9994 on the same test set. The no-tumor probability threshold of 0.95 was selected after inspecting that test set; the resulting reduction from 20 to 4 missed tumor slices and specificity of 0.993 are presented only as exploratory threshold behavior.

### 3.2 Comparison with specialist models and computational cost

Against specialist models, the unified network traded task scope, accuracy and efficiency (Tables 4–6). Resolution-matched mean Dice differences between MedNeXt and nnU-Net were mixed, and no endpoint had an exploratory Holm-adjusted *p*-value below 0.05 (Figure 4). The classification comparisons likewise remain exploratory because reliable patient clusters are unavailable. The results do not establish equivalence or architectural superiority.

**Table 4:** Comparison with specialist baselines.

Model/comparison	Task	Key result
MedNeXt-B 2D	Seg+Cls	Pooled mean Dice 0.7941; WT 0.8290; Acc 0.9648; AUC 0.9984
nnU-Net v2	Seg	Pooled mean Dice 0.8014; WT 0.8220
ResNet50	Cls	Acc 0.9839; exact McNemar *p*=0.0005; men AUC *p*=0.007
ConvNeXt-Tiny	Cls	Acc 0.9789; exact McNemar *p*=0.003; men AUC *p*=0.071 (n.s.)
SwinV2-Tiny	Cls	Acc 0.9698; exact McNemar *p*=0.383 (n.s.); men AUC *p*=0.011

**Table 5:** Resolution-matched arithmetic mean Dice in a common 512-pixel space. Mean differences and their intervals come from unrounded per-case values and may therefore differ by one unit in the last displayed digit from the difference of the rounded columns.

512-space Dice	nnU-Net mean	MedNeXt mean	Mean difference (M−N)	90% Bootstrap interval	Exploratory Holm-adjusted paired-rank result
Glioma	0.7537	0.7496	−0.0040	−0.0166 to +0.0083	Adjusted *p* ≥ 0.05 (exploratory)
Meningioma	0.8249	0.8657	+0.0408	+0.0204 to +0.0627	Adjusted *p* ≥ 0.05 (exploratory)
Pituitary	0.8391	0.8305	−0.0087	−0.0184 to +0.0007	Adjusted *p* ≥ 0.05 (exploratory)
Whole tumor	0.8220	0.8388	+0.0168	+0.0088 to +0.0250	adjusted *p* ≥ 0.05 (exploratory)

**Table 6:** Current device-matched efficiency, single forward pass at batch size 1.

Model/setting	Input	Params (M)	GMACs	Latency (ms)	Peak memory (MB)
MedNeXt-B 2D	256	10.58	14.44	4.44	155
nnU-Net v2	256	46.33	14.98	4.05	266
ResNet50	256	23.52	5.37	3.35	121
MedNeXt-B 2D	512	10.58	58.11	7.32	469
nnU-Net v2	512	46.33	59.94	4.61	480
ResNet50	512	23.52	21.47	3.35	164
Unified saving vs. two-model pipeline	Matched 256	84.9%	29.0%	40.1%	Not additive
Unified saving vs. two-model pipeline	Matched 512	84.9%	28.6%	8.1%	Not additive
Unified saving vs. two-model pipeline	Native deployment	84.9%	77.9%	44.2%	Not additive

GMACs, giga multiply-accumulate operations (10^9^ multiply-accumulate operations). Do not relabel these counts as FLOPs.

**Figure 4:**
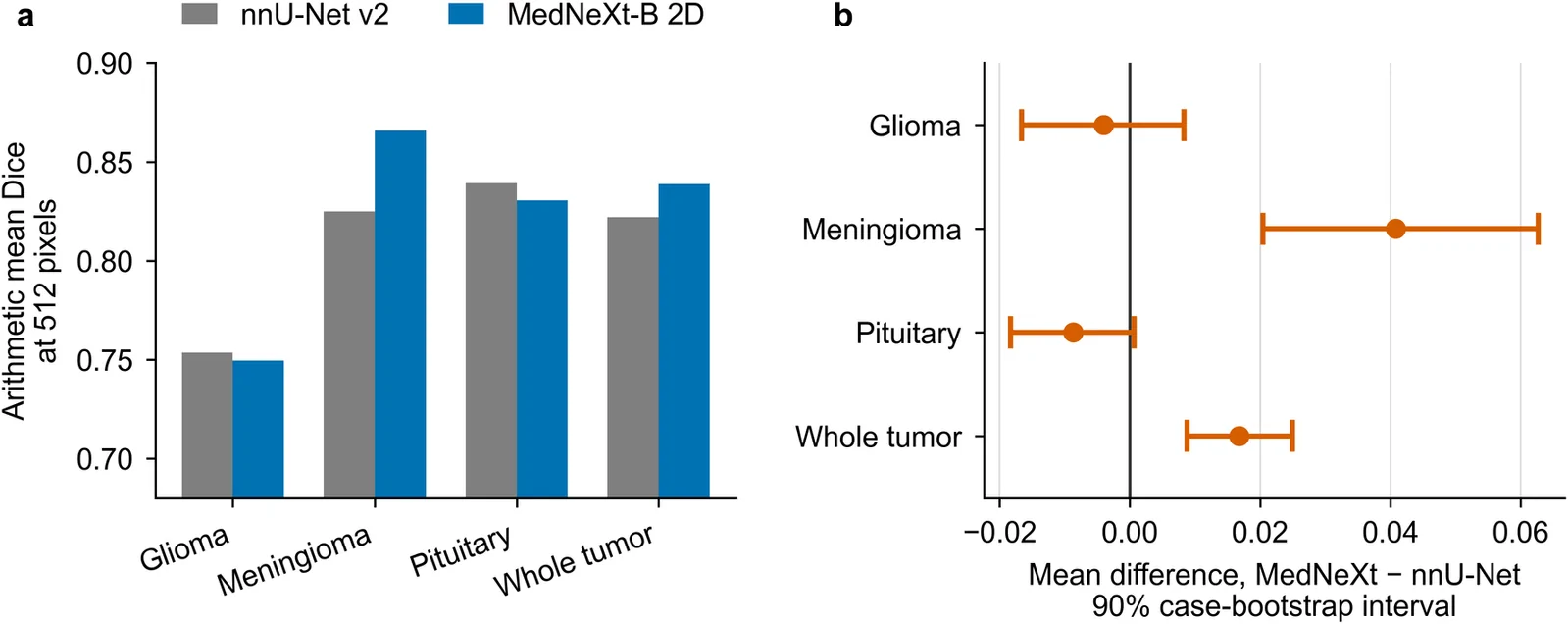
Resolution-matched segmentation comparison in a common 512-pixel space. **(a)** Arithmetic mean Dice per endpoint for nnU-Net v2 and MedNeXt-B 2D. **(b)** MedNeXt-minus-nnU-Net mean differences with 90% case-bootstrap intervals. The distributions were skewed by empty-mask failures; the figure does not claim equivalence.

The two segmentation models differ most in empty-mask frequency. At native resolution, nnU-Net returned an empty mask on 20/856 slices, compared with 6/856 for multi-task MedNeXt and 4/856 for segmentation-only MedNeXt. Because finite-case HD95 can favor a model by omitting these failures, the main MedNeXt estimate of 8.343 pixels is accompanied by the image-diagonal penalty sensitivity estimate of 10.823 pixels over all 856 masked slices (95% slice-bootstrap CI 8.807–13.107).

Device-matched benchmarking used one NVIDIA GeForce RTX 5090, PyTorch 2.7.1+cu128, a batch size of 1, 10 warm-up iterations, and 50 timed iterations (Table 6; Figure 5). Against sequential execution of nnU-Net followed by ResNet50, the unified model reduced measured latency by 40.1% at 256 pixels and 8.1% at 512 pixels. These values assume serial model execution and separate forward passes. Parallel execution, overlapping data transfer, shared preprocessing, or another system’s implementation could change the latency difference; therefore, the measurements are implementation-specific rather than a general deployment guarantee.

**Figure 5:**
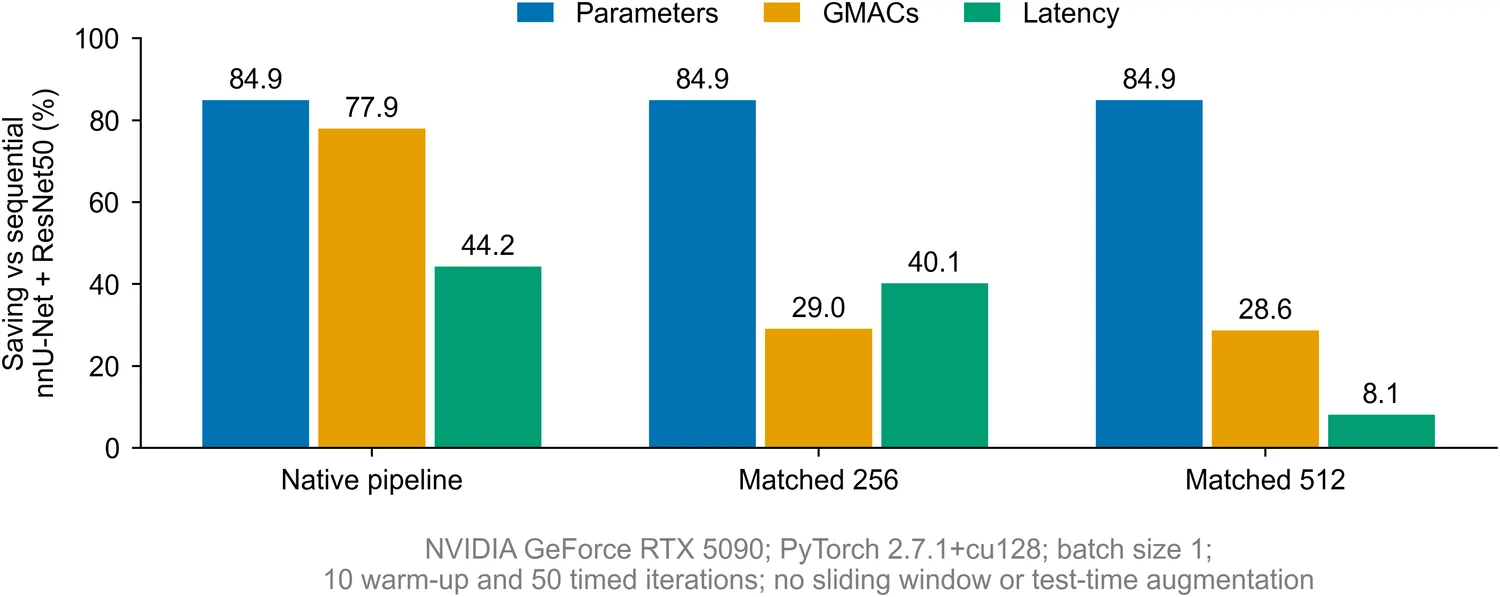
Device-matched efficiency on an NVIDIA GeForce RTX 5090 with PyTorch 2.7.1+cu128. Bars are the percentage saving of the unified model against the sequential nnU-Net-plus-ResNet50 pipeline, derived from single-forward-pass measurements at batch size 1 after 10 warm-up and 50 timed iterations (Table 6). Latency is hardware- and implementation-specific.

### 3.3 Joint versus single-task training and calibration

Joint and single-task training produced small, endpoint-dependent differences. The controls used the same MedNeXt backbone, split, schedule, and budget. Hodges–Lehmann estimates describe paired distributional shifts and do not equal arithmetic mean differences; all slice-level tests are exploratory because true patient grouping is unavailable.

In exploratory slice-level analyses, 8 of 10 paired distributional endpoints had Holm-adjusted *p*-values below 0.05; this thresholding is descriptive and non-confirmatory because the multiplicity family was not preregistered and patient clustering remains unresolved. In the separate true-class-only arithmetic-mean analysis, paired case-bootstrap 90% confidence intervals for multi-task minus single-task Dice were +0.0279 (0.0133–0.0435) for glioma, −0.0015 (−0.0154–0.0120) for meningioma, +0.0066 (−0.0034–0.0164) for pituitary and +0.0057 (0.0014–0.0102) for whole tumor. These endpoint-specific exploratory summaries neither establish clinical benefit nor rule out negative transfer (Table 7).

**Table 7:** Multi-task versus single-task paired comparison.

Endpoint (paired complete cases; *n* differs by endpoint; exploratory)	Multi-task	Single-task	Hodges–Lehmann difference; exploratory Holm-adjusted p
Glioma Dice (*n* = 258)	0.7190	0.6916	+0.0052, *p* = 0.0009
Pituitary Dice (*n* = 303)	0.8247	0.8182	+0.0063, *p* < 0.0001
Whole-tumor Dice (*n* = 856)	0.8290	0.8232	+0.0033, *p* < 0.0001
Per-case mean foreground Dice (*n* = 856)	0.8127	0.7966	+0.0039, *p* < 0.0001
Meningioma Dice (*n* = 306)	0.8557	0.8572	+0.0007, *p* = 0.034 (Hodges–Lehmann; the arithmetic mean difference is −0.0015)
Whole-tumor HD95 (pixels, *n* = 849)	8.300	8.755	−0.049, *p* = 0.0009
Pituitary HD95 (pixels, *n* = 297)	4.670	5.057	−0.133, *p* < 0.0001
Per-case mean foreground HD95 (pixels, *n* = 826)	8.078	8.429	−0.062, *p* = 0.0005
Glioma HD95 (*n* = 241) and meningioma HD95 (*n* = 288)	-	-	Holm-adjusted *p* ≥ 0.05 (exploratory)
Classification accuracy	0.9648	0.9548	exact McNemar *b* = 15, *c* = 5, *p* = 0.041 (*n* = 995)

The unified model had numerically lower ECE than pretrained ResNet50 and ConvNeXt-Tiny under equal-width bins, whereas equal-mass results varied and ResNet50 had a lower Brier score. Pseudo-patient-cluster bootstrap intervals were similar to slice-bootstrap intervals for the observed grouping, but that grouping has low recall. These comparisons are exploratory and do not establish a causal effect of pretraining or patient-level calibration superiority (Figure 6; Table 8).

**Figure 6:**
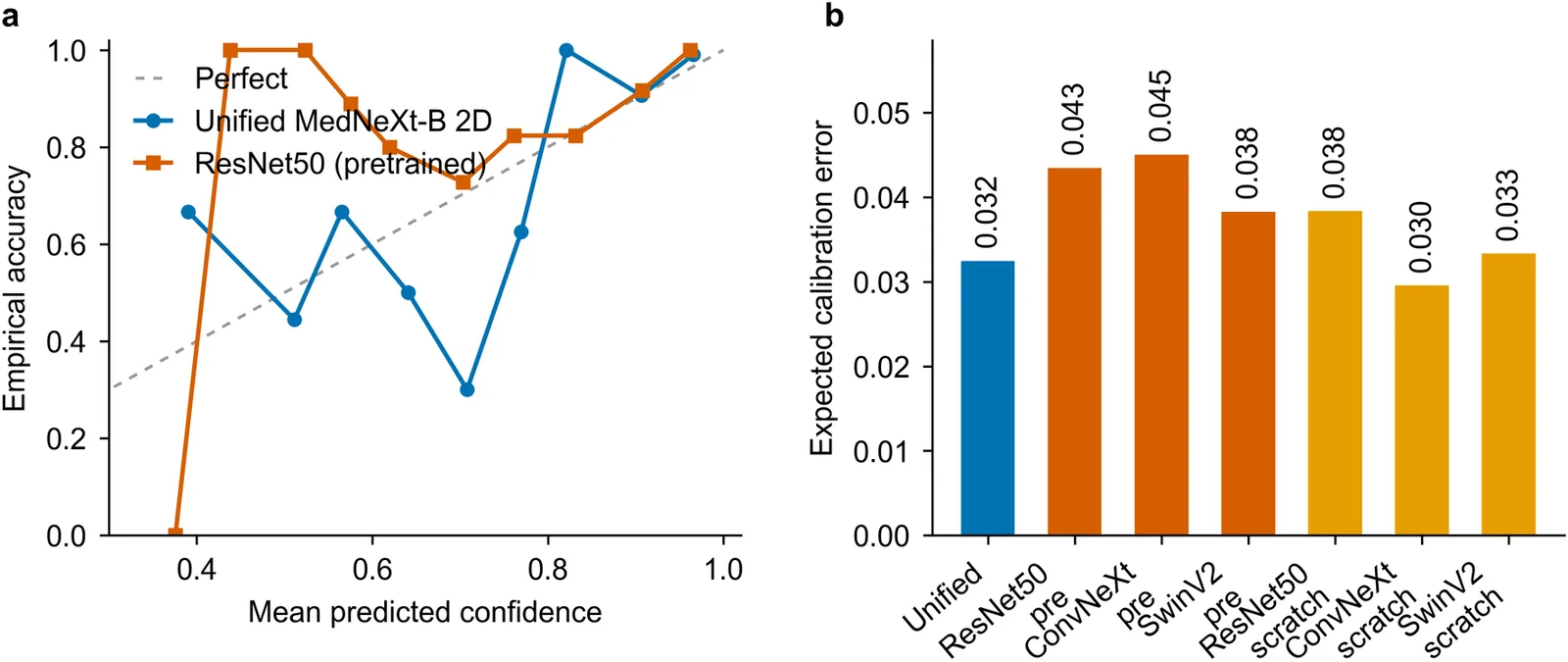
Internal-test classification calibration. (a) Reliability curves for the unified MedNeXt-B 2D model and pretrained ResNet50; the dashed diagonal indicates perfect calibration. (b) Expected calibration error for the unified model and ResNet50, ConvNeXt-Tiny, and SwinV2-Tiny classifiers, with the specialist classifiers evaluated both with ImageNet pretraining and with training from scratch.

**Table 8:** Classification calibration on the internal test set. Paired differences and their intervals are computed from unrounded values and may therefore differ by one unit in the last displayed digit from the difference of the rounded columns.

Metric	MedNeXt-B 2D	ResNet50	Exploratory paired difference (95% interval), *p*
ECE (15 equal-width bins)	0.0324	0.0435	Paired difference −0.0110 (−0.0217 to −0.0013), *p* = 0.021
ECE (equal-mass bins)	0.0292	0.0417	Paired difference −0.0124 (−0.0200 to 0.0015), *p* = 0.094; same direction, adjusted *p* ≥ 0.05 (exploratory)
Brier	0.0540	0.0319	ResNet50 lower (driven by higher accuracy)
vs. ConvNeXt-Tiny (pretrained)	0.0324	0.0451	Paired difference −0.0126 (−0.0225 to −0.0014), *p* = 0.026
vs. SwinV2-Tiny (pretrained)	0.0324	0.0383	Paired difference −0.0058 (−0.0161 to 0.0023), *p* = 0.119, n.s.

### 3.4 Single-factor algorithmic interventions

None of the six interventions had a higher value for the glioma Dice endpoint selected for this exploratory analysis. No dated protocol or preregistration documented the endpoint or multiplicity family, so all 48 tests are *post hoc* and exploratory. Effect sizes and adjusted *p*-values are shown only as descriptive analysis outputs, without confirmatory interpretation (Table 9; Figure 7).

**Table 9:** Glioma Dice endpoint selected for the *post hoc* exploratory analysis across six interventions.

Variant	Type	Delta glioma Dice	Raw p	Holm p	BH *p*
AG	structural	−0.00283	0.901	1.000	0.901
ASPP	structural	−0.00827	0.016	0.636	0.083
MS-FE	structural	−0.00761	0.017	0.677	0.083
HR-MSFE	structural	−0.00814	0.452	1.000	0.657
Focal Tversky	loss	−0.00514	0.005	0.227	0.042
Deep Supervision	supervision	−0.00128	0.256	1.000	0.490

**Figure 7:**
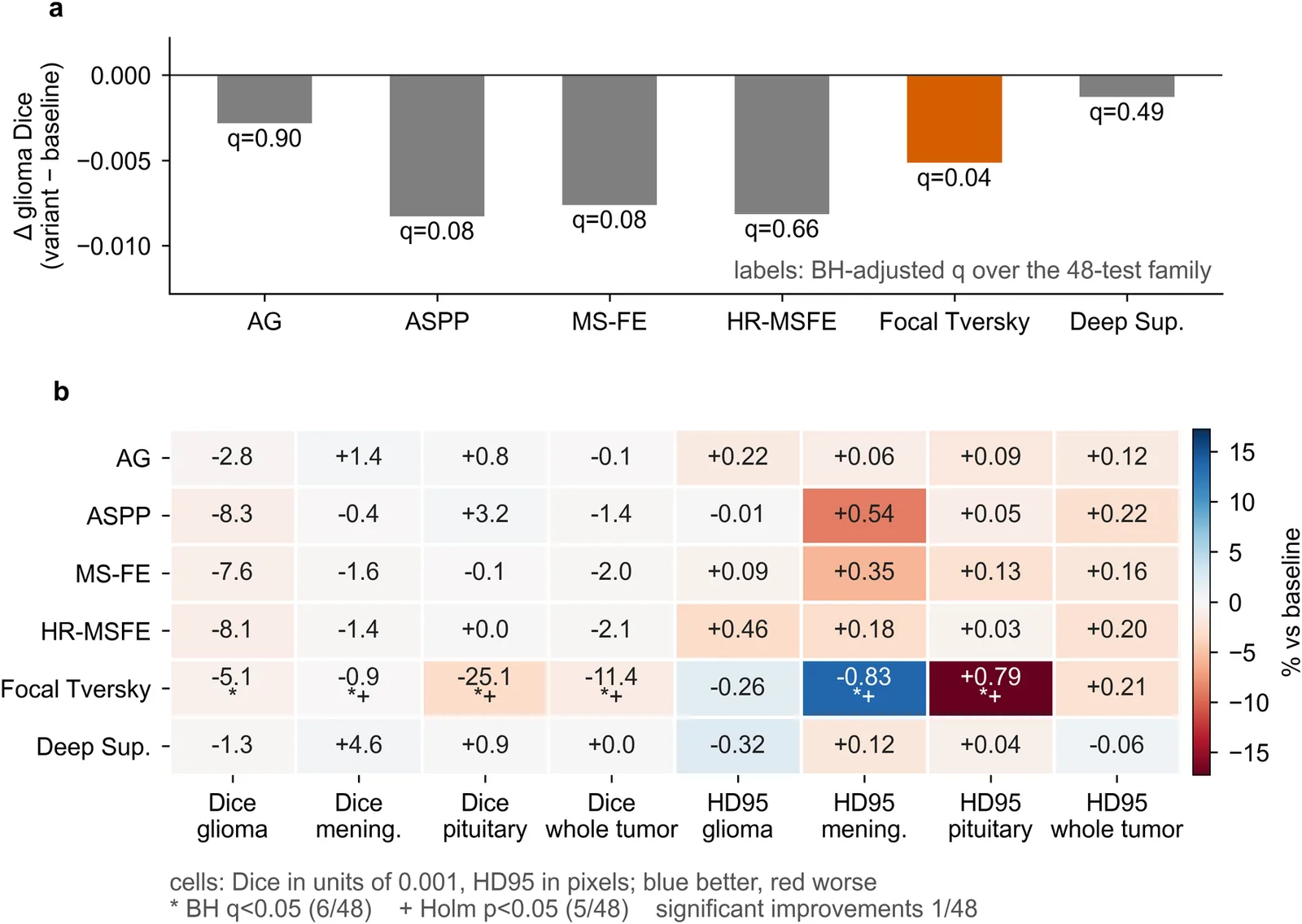
Exploratory single-factor interventions on the internal test set. **(a)** Change in the glioma Dice endpoint selected for this *post hoc* exploratory analysis; labels are Benjamini-Hochberg-adjusted q values over the 48-test family. **(b)** All six variants by eight segmentation metrics. These slice-level analyses were not preregistered and are not confirmatory.

### 3.5 Why small gliomas fail

Residual error was associated with small glioma lesions, and encoder geometry provides a plausible but unproven mechanism. Glioma Dice was higher in the largest lesion-area quartile (0.822) than in the smallest (0.582), while effective occupancy at the 16 × 16 bottleneck was low for many Q1 lesions (Figure 8). These observational analyses do not show that bottleneck resolution caused the errors, and the negative intervention results do not prove that lost detail cannot be recovered. Alternative explanations include annotation uncertainty, contrast, plane, optimization, and other correlated image characteristics. Figure 9 shows representative lesion-size overlays and examples with empty nnU-Net foreground predictions.

**Figure 8:**
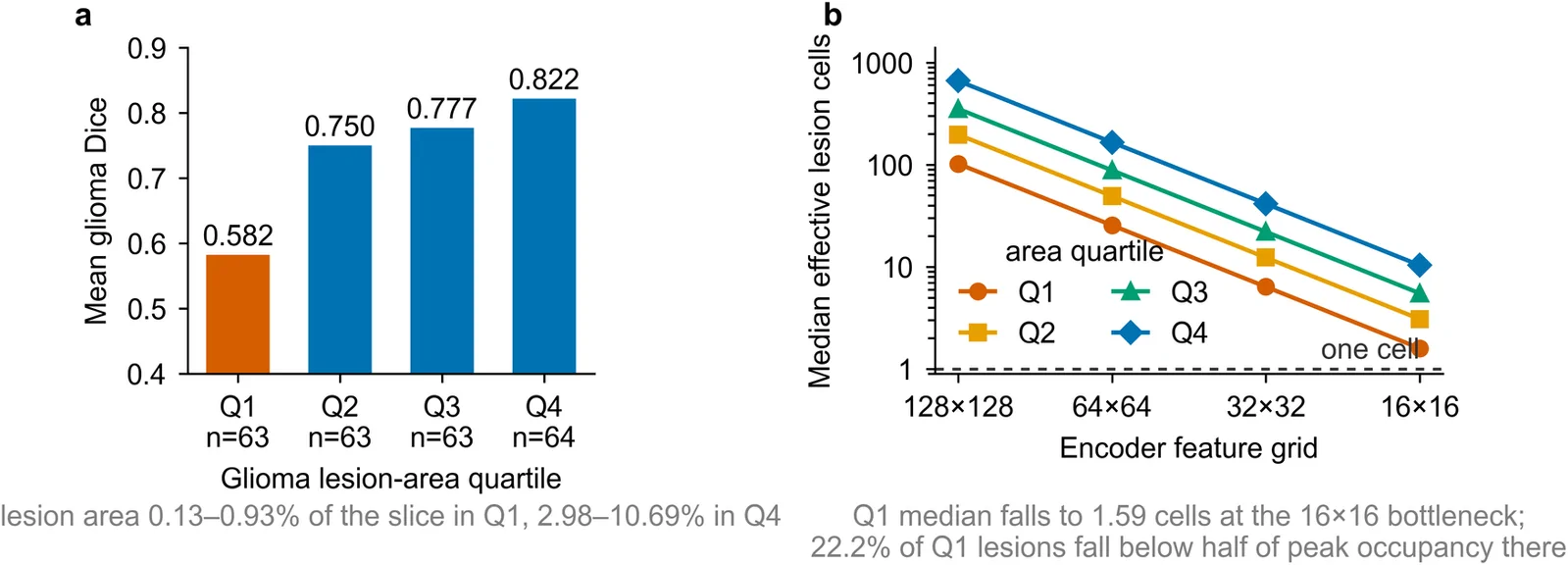
Association of glioma lesion size with segmentation performance and effective feature-grid occupancy. **(a)** Mean glioma Dice by lesion-area quartile. **(b)** Median effective lesion occupancy at each encoder feature grid, shown separately for the four lesion-area quartiles. The analysis illustrates a plausible mechanism and does not establish causality.

**Figure 9:**
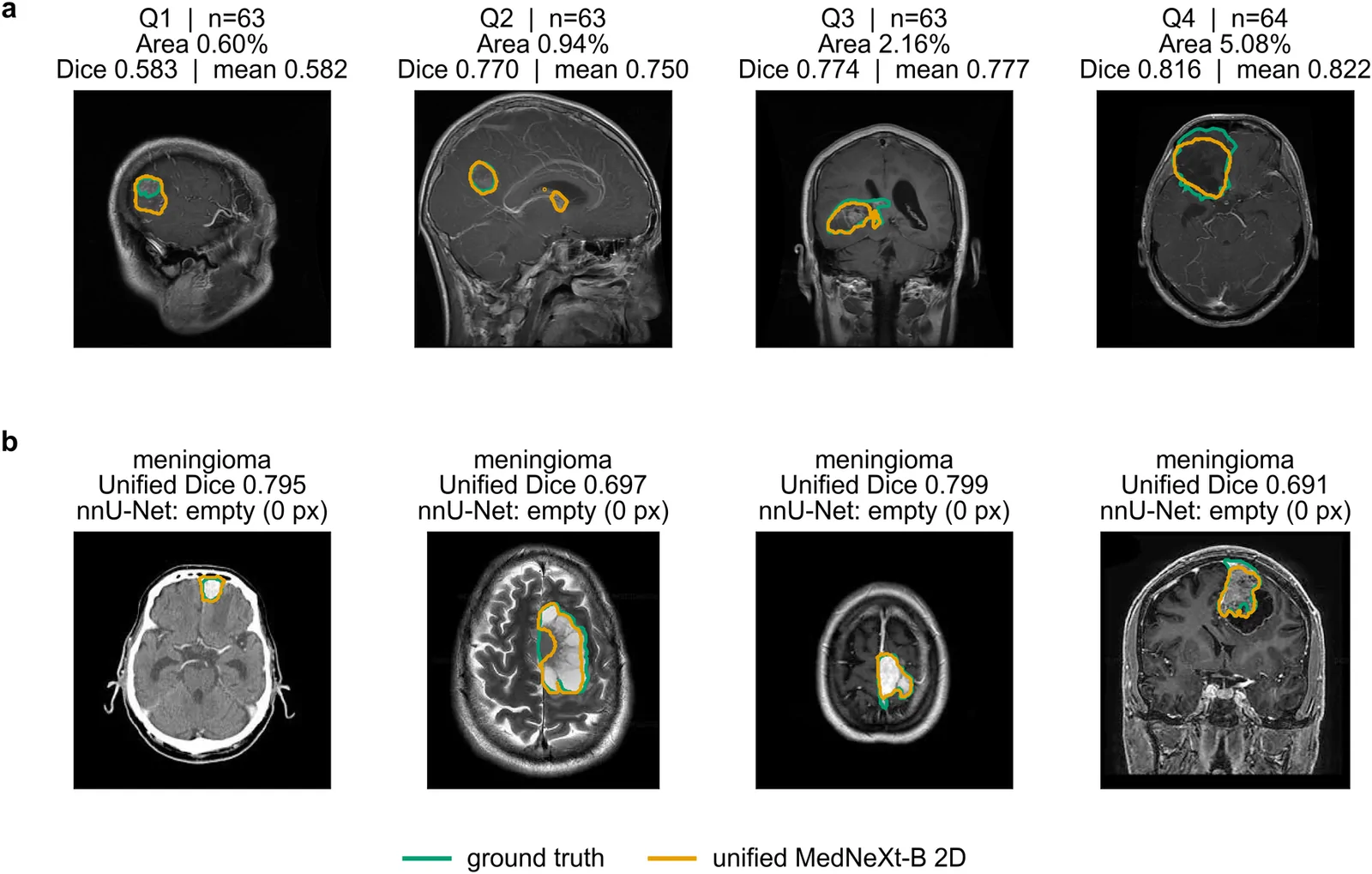
Qualitative overlays on the frozen internal test set. **(a)** One glioma case per lesion-area quartile, selected as the case whose Dice is closest to that quartile's mean Dice. **(b)** Four slices on which the BRISC-trained nnU-Net checkpoint produced an empty foreground mask. Green, ground truth; orange, unified MedNeXt-B 2D. The unified model predicts at 256 pixels and its mask is nearest-neighbour upsampled to the 512-pixel display grid for visualisation only; no reported metric is computed from these display images.

### 3.6 What the shared representation encodes

We used Grad-CAM as a *post hoc* attribution audit of the shared representation. The selected layer was the 512-channel 16 × 16 bottleneck, the last spatial tensor consumed by the classification head and the input to the decoder. For the ensemble-predicted class, channel weights were the spatial mean of the target-logit gradient; the weighted map was passed through ReLU, L1-normalized within each fold, averaged equally over five folds, and bilinearly upsampled to 256 × 256 with align_corners=false. Models were in evaluation mode with dropout disabled and EMA weights loaded. Because the head contains LayerNorm, GELU, dropout, and two linear layers after pooling, no equivalence to classical class activation mapping (CAM) is claimed. On 821 correctly classified masked tumor slices, mean pixel AUROC was 0.750 for glioma (95% slice-bootstrap CI 0.724–0.775), 0.554 for meningioma (0.521–0.588), and 0.705 for pituitary (0.679–0.731) (Figure 10). The analysis is exploratory, slice-level, and attributional; it does not explain or causally localize the decision.

**Figure 10:**
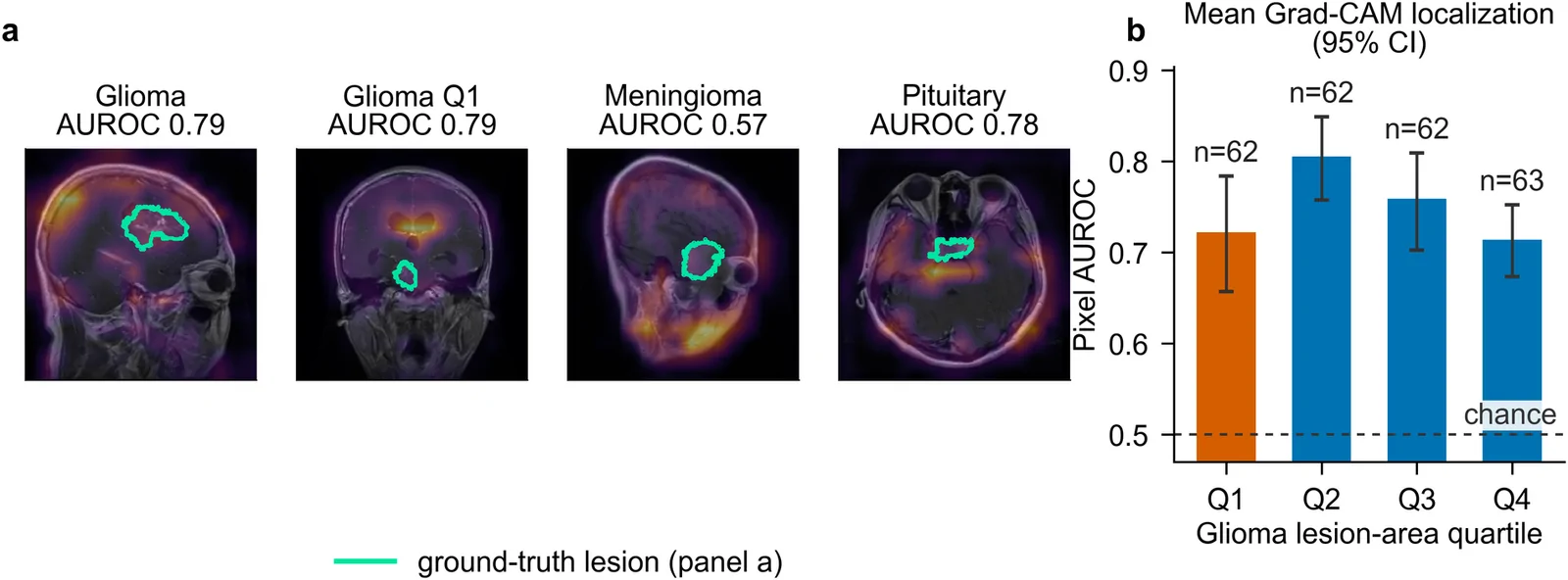
Grad-CAM attribution at the shared 512-channel 16 × 16 bottleneck. **(a)** Representative correctly classified masked slices; green outlines the annotated lesion. **(b)** Mean pixel-level lesion-localisation AUROC across glioma lesion-area quartiles with 5,000-resample slice-bootstrap 95% intervals.

### 3.7 Exploratory cross-dataset transfer evaluation

Exploratory cross-dataset transfer was poor in absolute terms. Zero-shot whole-tumor Dice was 0.274 on glioma, 0.445 on meningioma T1c, 0.258 on meningioma T2-FLAIR and 0.535 on pituitary (Table 10; Figure 11A). Differences in imaging sequence, preprocessing, label definitions and cohort construction preclude interpretation as clinical external validation or general architectural superiority.

**Figure 11:**
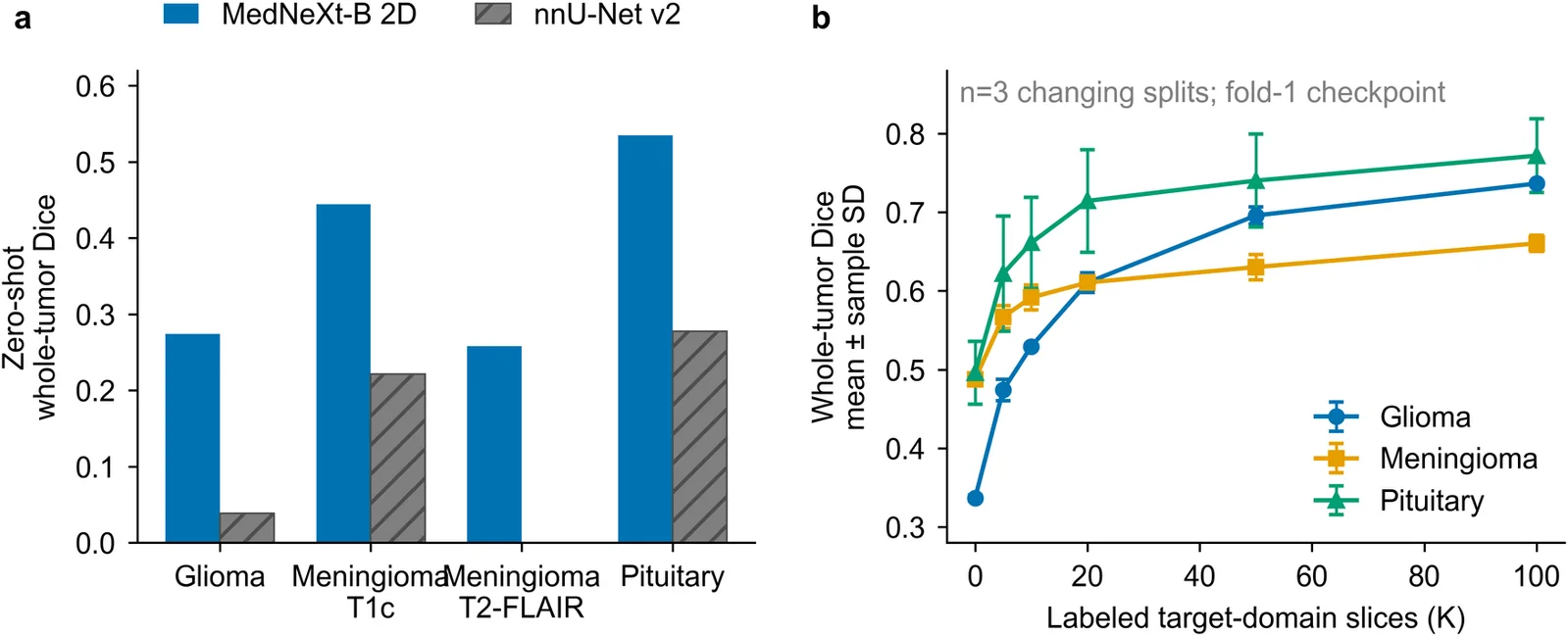
Exploratory cross-dataset transfer evaluation. **(a)** Zero-shot whole-tumor Dice on public converted-slice cohorts. **(b)** Few-shot descriptive summaries across three changing adaptation/test splits using one fold-1 checkpoint; these are not clinical validation results or formal confidence intervals.

**Table 10:** Exploratory zero-shot cross-dataset whole-tumor Dice.

External cohort (masked n)	MedNeXt-B 2D (unified)	nnU-Net (specialist)	nnU-Net median Dice
Glioma (*n* = 484)	0.274	0.038	0.000
Meningioma-T1c (*n* = 1,000)	0.445	0.221	0.000
Meningioma-T2f (*n* = 1,000)	0.258	-	-
Pituitary (*n* = 128)	0.535	0.278	0.000

A small number of labeled target slices recovered much of that gap. Using the fold-1 checkpoint, mean Dice over three changing adaptation/test splits rose from 0.337 at *K* = 0 to 0.736 at *K* = 100 for glioma, from 0.488 to 0.660 for meningioma T1c, and from 0.496 to 0.740 at *K* = 50 and 0.772 at *K* = 100 for pituitary adenoma (Table 11; Figure 11B). Each seed changed the held-out sample composition, and *n* = 3 is small, so we report sample SDs and observed ranges instead of normal-quantile confidence intervals. The *K* = 0 values differ from the main zero-shot five-fold ensemble because this analysis used fold 1 alone and scored a fixed held-out subset of each cohort (*n* = 384 glioma, 900 meningioma T1c, and 28 pituitary adenoma) rather than the full cohort. Combined external four-class accuracy was 0.419 (675/1,612) across the three T1c cohorts (484 glioma, 1,000 meningioma, and 128 pituitary adenoma), with no independent no-tumor class.

**Table 11:** Exploratory few-shot adaptation; descriptive *n* = 3 summaries.

External cohort	*K* = 0 mean	Selected adapted mean	Design note
Glioma	0.337	0.736 (*K* = 100)	*n* = 3 changing splits; fold−1 checkpoint
Meningioma T1c	0.488	0.660 (*K* = 100)	*n* = 3 changing splits; fold−1 checkpoint
Pituitary	0.496	0.740 (*K* = 50); 0.772 (*K* = 100)	*n* = 3 changing splits; fold−1 checkpoint
External four-class	accuracy 0.419 (675/1,612)	not evaluated	no independent no-tumor cohort

### 3.8 Size-matched near-duplicate contamination analysis

In the size-matched experiment, contaminated-minus-screened accuracy differences across seeds were +0.0281, +0.0281, +0.0211, +0.0281, and +0.0251 (mean +0.0261, sample SD 0.0031, range +0.0211 to +0.0281; indicative paired-seed t 95% confidence interval +0.0223 to +0.0300; primary hierarchical pseudo-cluster-and-seed 95% confidence interval +0.0207 to +0.0318). Mean class Dice differences were +0.0375, +0.0281, +0.0252, +0.0331 and +0.0338 (mean +0.0315, SD 0.0049, range +0.0252 to +0.0375; indicative t interval +0.0255 to +0.0376; primary hierarchical interval +0.0238 to +0.0394). Meningioma recall was higher by a mean difference of 0.0746 (SD 0.0055; primary hierarchical interval 0.0590–0.0911). For whole-tumor HD95, the mean paired-complete-case difference was −0.747 pixels across seeds (*n* = 847, 850, 849, 849, and 851; hierarchical interval −1.028 to −0.489); assigning the 362.04-pixel penalty to undefined values and retaining all 856 masked slices gave −2.270 pixels (−3.355 to −1.309). The same test-slice identifiers and validation folds were used in both conditions, and each training fold was matched in size and tumor-class-by-plane composition. These estimates quantify an association for a replacement-based sensitivity condition, not the original unscreened pool; other uncontrolled differences cannot be excluded, and the design does not prove image retrieval as the causal mechanism or patient-level leakage.

Positive differences denote higher accuracy, Dice, or recall in the replacement-based contaminated condition; negative HD95 differences denote lower distances. Hierarchical pseudo-cluster-and-seed intervals are the primary uncertainty summaries but do not establish patient-level independence. HD95 uses test-slice pairing and the complete-case rule stated in Methods; the all-masked penalty analysis is a sensitivity analysis.

The contamination, architectural, and multi-task experiments address different contrasts and, in several cases, different endpoints. We therefore report their effect sizes and uncertainty separately and do not rank them as definitively larger or smaller. No formal difference-between-effects test was performed. Table 12 summarizes endpoint-specific contamination differences for accuracy, mean class Dice, whole-tumor Dice, HD95, and class recall. Table 5 reports architecture-specific arithmetic mean Dice differences with paired case-bootstrap intervals, while the task-sharing section reports its own endpoint-specific paired summaries. These results provide descriptive context but are not presented as like-for-like or common-scale effect estimates.

**Table 12:** Exploratory multi-seed paired benchmark differences for the replacement-based contaminated condition (contaminated minus near-duplicate-screened).

Endpoint	Mean ± SD (range)	Indicative paired-seed t 95% interval (df=4)	Primary hierarchical cluster-seed 95% confidence interval
Accuracy	+0.0261 **±** 0.0031 (+0.0211 to +0.0281)	+0.0223 to +0.0300	+0.0207 to +0.0318
Mean class Dice	+0.0315 ± 0.0049 (+0.0252 to +0.0375)	+0.0255 to +0.0376	+0.0238 to +0.0394
Whole-tumor Dice	+0.0096 ± 0.0014 (+0.0074 to +0.0110)	+0.0078 to +0.0113	+0.0065 to +0.0128
Meningioma recall	+0.0746 ± 0.0055 (+0.0660 to +0.0792)	+0.0677 to +0.0814	+0.0590 to +0.0911
Whole-tumor HD95	−0.747 ± 0.194 (−0.947 to −0.466)	−0.988 to −0.506	−1.028 to −0.489

We treat the comparison between contamination and model modification as descriptive, not inferential, and did not perform a formal difference-between-effects test. The three experiment families differ in contrasts, endpoints, estimands and uncertainty procedures. The contamination effect is a size- and composition-matched contrast across five seeds. The architectural experiments are single-seed, single-factor segmentation contrasts. The task-sharing experiment compares matched single-task and multi-task models but uses its own endpoint-specific analyses. These differences preclude a common difference-of-differences estimand and comparable uncertainty estimates. We therefore removed cross-endpoint comparisons, including the earlier juxtaposition of classification accuracy with glioma Dice. We also avoid describing the remaining values as like-for-like or on a common scale. Contamination estimates are interpreted only within their named endpoints in Table 12; architectural and task-sharing estimates remain separate contextual analyses in Tables 5, 7, 9. None of these analyses are confirmatory, and we make no claim that one category of effect is statistically or generally larger than another.

## 4 Discussion

### 4.1 Principal findings

The size-matched multi-seed comparison showed consistently higher slice-level benchmark performance when detected test-linked near-duplicates were retained. The paired mean difference was +0.0261 for accuracy and +0.0315 for mean class Dice; the hierarchical intervals did not cross zero within the available pseudo-cluster analysis. This is a descriptive exploratory result, not confirmatory evidence: the replacement condition is not the original unscreened pool, constituent source matching was impossible, the screened arm may retain undetected related slices, and reliable patient identifiers were unavailable.

The practical implication is narrow: scores from pre-partitioned slice collections should be accompanied by reproducible exact-hash and near-duplicate screening rules. The effect magnitude reported here is specific to BRISC2025, the selected pHash threshold, the matched sampling procedure, and the tested model. It is not a universal correction factor and does not demonstrate how a true patient-level split would change performance.

Recent brain tumor MRI studies combine convolutional neural networks (CNNs), transformers, segmentation, classification and explainability, while the broader medical-imaging literature documents leakage and validation risks (18–20, 35–42). This study is not the first to use a joint model. Its contribution is a reproducible, size-matched multi-seed audit of detected near-duplicate contamination, together with explicit limitations on patient-level and cross-dataset interpretation.

### 4.2 The unified model against specialist models

The unified model earns its place as an efficient baseline. Sharing features cuts parameter storage and model-management overhead, and the paired controls give limited evidence against negative transfer in this dataset without isolating a causal representation mechanism. The architecture reads single slices, carries no cross-slice context, and leaves small-glioma and out-of-domain errors unresolved.

nnU-Net v2 brings mature self-configuration and a slightly higher native-resolution mean class Dice. Resolution-matched arithmetic means were mixed, and the experiment establishes no equivalence. Its BRISC-trained checkpoint produced more empty masks under the cross-domain test we ran, but zero-shot use without target reconfiguration falls outside nnU-Net's intended workflow.

ImageNet-pretrained classifiers gave stronger internal classification accuracy and, in some cohorts, stronger external recall. They need a separate segmenter, and lower classification error did not consistently mean lower ECE. The from-scratch control was not retuned and serves only as a sensitivity analysis of the pretraining confound.

The three approaches trade task scope, internal accuracy, calibration, parameter storage, and transfer behavior, and no single ranking covers them (Figure 12). The evidence supports continued technical evaluation and not clinical deployment.

**Figure 12:**
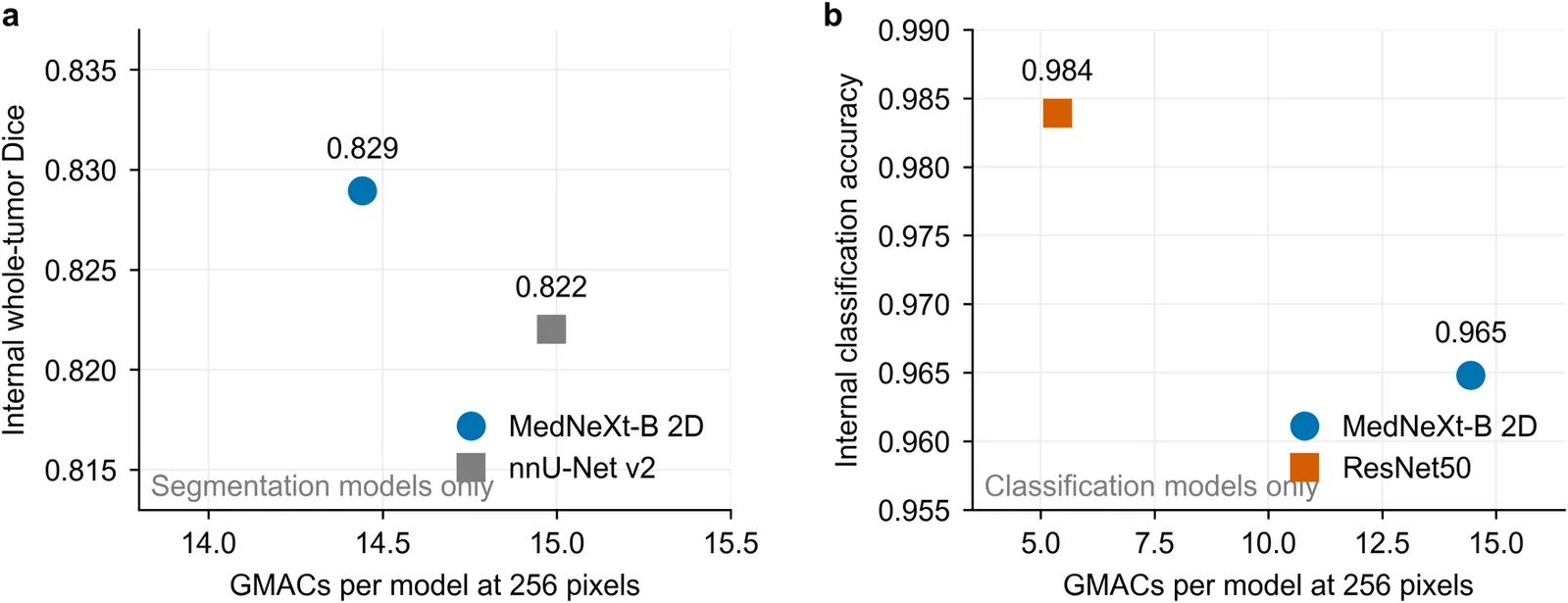
Internal efficiency–accuracy trade-off at a matched 256-pixel input. **(a)** Whole-tumor Dice against per-model GMACs for the two segmentation models. **(b)** Classification accuracy against per-model GMACs for the two classifiers. Compute values are those of Table 6; the comparison is not a clinical utility analysis.

Efficiency values are single-forward-pass measurements. Reported pipeline latency assumes sequential execution of the segmentation and classification specialists. Parallel execution, shared preprocessing, batching, and hardware scheduling may change the realized latency difference; the reported values are therefore specific to the benchmarked implementation.

### 4.3 Calibration and domain shift

The unified model had numerically lower ECE than two pretrained classifiers, but these slice-level comparisons are exploratory. Cross-dataset transfer used heterogeneous public converted-slice cohorts and does not constitute independent clinical external validation. The observations motivate target-domain evaluation and domain-adaptation strategies (43) without establishing that multi-task learning caused greater robustness.

### 4.4 Few-shot adaptation

The few-shot curves are exploratory and do not represent a validated deployment pathway. Adaptation and test composition changed by seed, one fold model was used, and no independent patient-level test set was fixed. A confirmatory experiment should use verified patient identifiers, a fixed external test cohort, independently varied adaptation samples and optimization seeds, and uncertainty quantification over patients and training runs.

### 4.5 Limitations

Several limitations bound these conclusions. BRISC provides no true patient identifiers, and the pHash-derived grouping recovers only 5.8% of known same-patient slice pairs in an independent calibration dataset. The procedure is therefore low-recall near-duplicate screening, not leakage control or patient separation, and the screened arm may retain undetected related slices. Residual overlap could attenuate the observed contrast if it acts monotonically in the same direction, but the overall bias direction is not identifiable because other composition differences, including constituent source, remain uncontrolled. The replacement-based contaminated condition is not the original 4,955-image unscreened pool. The direction and magnitude of performance under a stricter patient-level exclusion are also unknown because additional exclusions would remove both possible leakage and legitimate training information; no lower-bound claim is made. The comparison was matched on training size and tumor class by imaging plane, but per-image constituent source provenance was unavailable. Five seeds improve characterization of training variability but remain a modest sample; their Student-t intervals are indicative, while the primary hierarchical intervals account only for those seeds and detectable pseudo-clusters and cannot establish patient-level independence. Slice-level McNemar, Wilcoxon, and DeLong tests, multiplicity analyses, the 0.95 threshold, Grad-CAM, and few-shot analyses are *post hoc* and exploratory. The maximum-diagonal HD95 sensitivity analysis reduces, but does not remove, dependence on the chosen empty-case penalty. Augmentation plausibility was not clinically adjudicated. Cross-dataset cohorts differ in imaging sequence, preprocessing, and labels and contain no independent no-tumor cohort.

## 5 Conclusion

In a size- and tumor-class-by-plane-matched experiment across five seeds, the replacement-based contaminated condition had higher accuracy and mean class Dice, with mean differences of 0.0261 and 0.0315, respectively. These exploratory hierarchical intervals did not cross zero under the available pseudo-cluster grouping, but low screening recall, unavailable constituent source provenance, the use of only five seeds, and slice-level sampling prevent patient-level or causal interpretation. Other uncontrolled differences cannot be excluded. The MedNeXt-B 2D model remains an efficient research baseline for joint slice segmentation and classification. The work supports reproducible near-duplicate screening, transparent split reporting, and quantification of uncertainty across training runs; it does not support clinical deployment, patient-level validation or a definitive ranking of contamination and architectural effects.

## Data Availability

The datasets analyzed in this study are publicly accessible from their original providers under the applicable licences and access conditions: BRISC2025 (27), Medical Segmentation Decathlon Task01 BrainTumour (32), BraTS-MEN 2023 (33), and the pituitary MRI dataset (34). A permanent Zenodo record is available at https://doi.org/10.5281/zenodo.22205980. The record contains documentation describing the dataset provenance, recorded software environment, split-construction procedure, pHash graph rule and Hamming-distance threshold, exclusion logic, five-fold sample sizes, matched-contamination design, principal five-seed results, local integrity checks, and known limitations.
