# Supplementary material for: Near-duplicate contamination inflates brain MRI benchmark performance: a size-matched, multi-seed MedNeXt-B study
Source: Front Med (Lausanne). 2026 Oct 8;13:1955863. doi: 10.3389/fmed.2026.1955863 (PMC13690018; doi:10.3389/fmed.2026.1955863)
Supplement: Supplementary file 1 [file Datasheet1.pdf]

frontiers

Supplementary Material

Near-Duplicate Contamination Inflates Brain MRI Benchmark Performance: A Size-Matched, Multi-Seed MedNeXt-B Study

Pseudocode, screening calibration, uncertainty analyses, and dataset composition

This supplement provides implementation details and Tables S1-S6. The pseudocode is a schematic description of the reported computations, not a standalone executable software package. References to numbered figures, tables, sections, and references of the main text follow the accompanying manuscript. The internal unit of analysis is the two-dimensional image (slice), not a verified patient.

S1. Unified MedNeXt-B 2D multi-task model

The shared MedNeXt-B encoder feeds both the native segmentation decoder and a bottleneck classification head. One forward pass returns four-channel segmentation logits and four-class classification logits; masks and probabilities are derived during evaluation. The model reuses official MedNeXt modules through a custom multi-task wrapper; the imported backbone modules are not edited. This is the study-specific two-dimensional implementation, rather than the official training protocol (main-text Section 2.3 and Figure 2).

```
class MedNeXtMultiTask:
    # Official MedNeXt-B modules: 2D, group normalization, base width 32.
    cls_head = Sequential(
        AdaptiveAvgPool2d(1), Flatten(), LayerNorm(512),
        Dropout(0.3), Linear(512, 256), GELU(),
        Dropout(0.3), Linear(256, 4))

    def encode(x):                          # x: [B, 1, 256, 256]
        x = backbone.stem(x)
        r0 = backbone.enc_block_0(x); x = backbone.down_0(r0)
        r1 = backbone.enc_block_1(x); x = backbone.down_1(r1)
        r2 = backbone.enc_block_2(x); x = backbone.down_2(r2)
        r3 = backbone.enc_block_3(x); x = backbone.down_3(r3)
        bott = backbone.bottleneck(x)     # 512 channels, 16 x 16
        return (r0, r1, r2, r3, bott)

    def decode(feats):                    # native decoder and skips
        r0, r1, r2, r3, x = feats
        x = backbone.dec_block_3(r3 + backbone.up_3(x))
        x = backbone.dec_block_2(r2 + backbone.up_2(x))
        x = backbone.dec_block_1(r1 + backbone.up_1(x))
        x = backbone.dec_block_0(r0 + backbone.up_0(x))
        return backbone.out_0(x)          # [B, 4, 256, 256]

    def forward(x):
        feats = encode(x)
        seg_logits = decode(feats)
        cls_logits = cls_head(feats[-1])  # original bottleneck
        return {seg_logits, cls_logits}
```

BRISC supplies binary tumor masks. Background pixels have label 0, while foreground pixels receive the image class label: glioma 1, meningioma 2, or pituitary 3. No-tumor images and images without a supplied mask have an all-zero placeholder target and has_mask=false. They contribute to classification loss only, not to segmentation loss or segmentation metrics. Segmentation evaluation is restricted to the 856 supplied masks. The two task weights are both 1.

```
# Schematic baseline objective; apply the mask-availability filter first.
m = batch.has_mask
loss_seg = 0
if any(m):
    loss_seg = 0.5 * Dice(seg_logits[m], target[m])
    loss_seg += 0.5 * CE_seg(seg_logits[m], target[m])
loss_cls = CE_cls(cls_logits, batch.cls, label_smoothing=0.05)
loss = loss_seg + loss_cls
```

S2. Attention Gate (AG) - decoder skips

Inserted at the four decoder skips; the skip and gating tensors have the same channels and spatial size, so no resampling is used. Zero-initializing psi produces a spatially uniform alpha=0.5 and initially scales every skip by one half. This initialization is symmetric but is not an identity mapping.

```
class AttentionGate(channels):            # inter = channels // 2
    theta = Conv1x1(channels -> inter)
    phi = Conv1x1(channels -> inter)
    norm = GroupNorm(inter)
    psi = Conv1x1(inter -> 1)              # weight and bias = 0
    def forward(skip, gate):              # equal [B, C, H, W] shapes
        a = ReLU(norm(theta(skip) + phi(gate)))
        alpha = sigmoid(psi(a))           # initially uniform 0.5
        return skip * alpha
```

S3. ASPP - bottleneck multi-scale context (segmentation path only)

Applied to the 512-channel, 16x16 bottleneck on the segmentation decode path. Within the same forward pass, the classification head reads the unmodified bottleneck tensor. The fusion convolution is zero-initialized, so the ASPP module is an exact identity mapping at initialization.

```
class ASPP(channels, rates=(2, 3)):         # inter = channels // 4
    b0 = Conv1x1(channels -> inter)
    b_dil = [Conv3x3(channels -> inter, dilation=r, padding=r)
             for r in rates]
    b_pool = Seq(AdaptiveAvgPool2d(1), Conv1x1(channels -> inter))
    fuse = Conv1x1(inter * 4 -> channels)  # weight and bias = 0
    norm, act = GroupNorm(channels), GELU()
    def forward(x):                       # [B, 512, 16, 16]
        feats = [b0(x)] + [b(x) for b in b_dil]
        p = upsample(b_pool(x), size=x.hw)
        y = act(norm(fuse(concat(feats + [p]))))
        return x + y                     # y = 0 at initialization
```

S4. MS-FE - spatially gated multi-scale residual (bottleneck)

Combines additive multi-scale context with a learned spatial gate: out = x + sigmoid(saliency(x)) * MultiScale(x). The gate is not supervised as a foreground mask and is therefore interpreted only as a learned spatial weight. The projection is zero-initialized, so MS-FE is an identity mapping at initialization. It is used only on the segmentation decode path.

```
class MSFE(channels, rates=(2, 3)):         # inter = channels // 4
    b0, b_dil, b_pool = (as in ASPP)
    proj = Conv1x1(inter * 4 -> channels)  # weight and bias = 0
    norm, act = GroupNorm(channels), GELU()
    saliency = Conv1x1(channels -> 1)
    def forward(x):
        feats = [b0(x)] + [b(x) for b in b_dil]
        feats += [upsample(b_pool(x), x.hw)]
        ctx = act(norm(proj(concat(feats))))
        a = sigmoid(saliency(x))          # learned [B, 1, H, W] gate
        return x + a * ctx               # equals x at initialization
```

S5. High-resolution multi-scale feature enhancement (HR-MSFE)

High-resolution multi-scale feature enhancement (HR-MSFE) is the high-resolution-skip variant of the multi-scale enhancement module described in Section S4. It uses the same MSFE module class as the bottleneck variant but places it on the 128-channel, 64x64 r2 skip instead of the 512-channel, 16x16 bottleneck. The relocation consequently changes channel width, parameter count, and computation as well as spatial resolution. It is applied only on the segmentation decode path. At fixed shared weights, the classification branch input and operations are unchanged.

```
class MedNeXtMultiTaskHRMS(MedNeXtMultiTask):
    hrms = MSFE(n_channels * 4)            # MSFE(128), 64 x 64 skip
    def decode(feats):
        r0, r1, r2, r3, x = feats
        x = backbone.dec_block_3(r3 + backbone.up_3(x))
        r2 = hrms(r2)                      # segmentation path only
        x = backbone.dec_block_2(r2 + backbone.up_2(x))
        x = backbone.dec_block_1(r1 + backbone.up_1(x))
        x = backbone.dec_block_0(r0 + backbone.up_0(x))
        return backbone.out_0(x)
# The classification head reads the original bottleneck in the same pass.
```

S6. Focal Tversky loss (FTL) - region loss replacement

Under the fixed task-weighting configuration used in this experiment, the segmentation cross-entropy term, classification cross-entropy term, coefficients, and has_mask handling follow the baseline computational path; a separate loss class replaces Dice with focal Tversky as the region term. Because a=0.7 exceeds b=0.3, a false-negative contribution of a given magnitude is weighted more than a false-positive contribution. The implementation raises (1-TI) to the exponent 0.75. This is numerically equivalent to gamma=4/3 under the original Abraham-Khan (1-TI)^(1/gamma) convention. This study did not independently establish that this exponent specifically focuses small or hard cases (main-text reference 25).

```
def FocalTversky(logits, target, a=0.7, b=0.3, gamma=0.75, s=1.0):
    p = softmax(logits, dim=1); y = one_hot(target)
    TP = sum(p * y)                        # over batch and spatial axes
    FN = sum((1 - p) * y)
    FP = sum(p * (1 - y))
    TI = (TP + s) / (TP + a * FN + b * FP + s)
    return mean_over_foreground_classes((1 - TI)**gamma)
# Only has_mask=true images enter FocalTversky and CE_seg.
# L = 0.5 * FocalTversky + 0.5 * CE_seg + CE_cls
# CE_cls uses every image with label_smoothing=0.05; both task weights = 1.
```

S7. Deep Supervision (DS) - multi-scale training supervision

The DS model exposes segmentation logits at five decoder scales (256, 128, 64, 32, and 16 pixels). During training, the region and cross-entropy losses are applied at every scale against the ground-truth mask resized by nearest-neighbor interpolation. Geometric weights w_s=(1/2^s) are normalized to sum to one. As in the baseline, only images with has_mask=true contribute to the segmentation terms. Inference and reported metrics use only the full-resolution seg_logits output, preserving the baseline output and evaluation protocol. Training nevertheless adds four auxiliary heads and their losses, so the DS variant is not architecturally identical to the baseline.

```
def MultiTaskDeepSupervision(outputs, batch):
    ds_list = outputs.ds_list            # [out_0, ..., out_4]
    w = normalize([1 / 2**s for s in range(len(ds_list))])
    m = batch.has_mask
    loss_seg = 0
    if any(m):
        for s, seg_s in enumerate(ds_list):
            gt_s = nearest_downsample(batch.mask[m], seg_s.hw)
            loss_seg += w[s] * (0.5 * Dice(seg_s[m], gt_s)
                                + 0.5 * CE_seg(seg_s[m], gt_s))
    loss_cls = CE_cls(outputs.cls_logits, batch.cls,
                      label_smoothing=0.05)
    return loss_seg + loss_cls           # inference uses out_0 only
```

S8. External-data manifest (scope and reproducibility)

Table S1 lists the cohorts used for zero-shot evaluation. These zero-shot samples were not used in BRISC model training, tuning, or internal model selection. The separate few-shot analyses in the main text used designated external adaptation subsets; within each run, those subsets were disjoint from the corresponding held-out external test subset. These converted-slice cohorts differ in sequence, preprocessing, and label definitions and are not an independent patient-level clinical validation cohort (main-text Sections 2.9 and 3.7). No independent no-tumor cohort passed the overlap screen, so external four-class specificity and AUC are not estimable.

For each zero-shot cohort, the retained machine-readable manifest records the cohort, class, evaluated image and mask counts, local path, and an aggregate SHA-256 computed over sorted filename:hash entries. Source publications are identified in the main manuscript references, but source URLs and download dates are not fields in this manifest. The public pituitary resource contains 136 patients. The conversion log recorded 135 labeled cases after one no-label exclusion, whereas the retained evaluated set contains 128 cases. The aggregate hash pins those 128 cases, but a contemporaneous exclusion record for the other seven converted cases was not retained.

Table S1. External cohorts and protocol-specific use.

Cohort	Class	Evaluated images = masks	Source
MSD Task01 BrainTumour	Glioma	484	Medical Segmentation Decathlon (BraTS); main-text reference 32
BraTS-MEN 2023 (T1c)	Meningioma	1,000	ASNR-MICCAI BraTS-MEN 2023; main-text reference 33
BraTS-MEN 2023 (T2f)	Meningioma	1,000	ASNR-MICCAI BraTS-MEN 2023; main-text reference 33
Pituitary PitNET (T1ce)	Pituitary	128	Pandit et al., Scientific Data (2025), doi: 10.1038/s41597-024-04218-8; main-text reference 34

Counts refer to the evaluated image-mask examples in the converted-slice cohorts, not independently validated patient-level outcomes. T1c/T1ce, contrast-enhanced T1; T2f, T2-FLAIR. The separate few-shot analysis used one fold-1 checkpoint and three changing adaptation/test splits, rather than the full-cohort five-fold zero-shot ensemble.

S9. Qualitative small-lesion overlays (Figure 9 of the main text)

Figure 9A of the main text shows one glioma case from each lesion-area quartile, selected as the case with Dice closest to that quartile's mean. These panels describe how the observed errors changed with lesion area; they do not establish a causal failure mechanism. In the smallest quartile, the predicted-to-annotated foreground-area ratio averaged 1.84 (median 1.09), 57% of cases over-segmented, and mean precision (0.561) was below mean recall (0.664). These averages indicate that over-segmentation contributed more than under-coverage in that quartile. In the largest quartile, the area ratio was 0.85 and mean precision (0.904) exceeded mean recall (0.776). The 16x16 bottleneck is spatially coarse, but the decoder also receives higher-resolution skip features. The observations are therefore consistent with, but do not prove, an effect of bottleneck dilution.

Figure 9B shows four examples among the 20 slices on which the BRISC-trained nnU-Net checkpoint returned an empty foreground mask while the unified model did not. These empty predictions contributed materially to the positive arithmetic-mean differences for meningioma and whole-tumor Dice, but they do not explain every between-model difference. The unified mask is predicted at 256 pixels and nearest-neighbor upsampled to the 512-pixel display grid for visualization only; no reported metric is computed from these display images.

Table S2. Design, exploratory hypotheses, and interpretation boundaries of the six single-factor interventions.

Variant	Insertion / change	Algorithmic hypothesis	Interpretation boundary
AG	Four decoder skips	Filter skip background and refine boundary fusion.	Changes in whole-tumor Dice or HD95 do not establish an improvement in the selected glioma Dice endpoint.
ASPP	512-channel, 16x16 bottleneck	Provide multi-scale atrous context for tumor scale.	A low-resolution context module does not by itself establish recovery of small-lesion detail.
MS-FE	512-channel, 16x16 bottleneck	Assess a spatially gated multi-scale residual for small-lesion response.	The learned gate is not a supervised foreground mask; improved glioma Dice requires empirical evidence.
HR-MSFE	128-channel, 64x64 skip	Assess multi-scale enhancement at a higher-resolution skip.	The change also alters channel width and computation. A non-significant result does not prove that lost detail cannot be recovered.
Focal Tversky	Segmentation region loss	Increase false-negative weighting and assess recall empirically.	This is a loss intervention, not a new architecture; effects on other-class Dice require separate assessment.
Deep Supervision	Multi-scale training supervision	Assess additional supervision for gradient flow and scale learning.	Training adds auxiliary heads; the full-resolution inference output and evaluation protocol are retained.

The input size, maximum epoch budget, optimizer, schedule, and data split match the baseline. Each variant implements one intended intervention family; associated changes include auxiliary deep-supervision heads and the channel-width change accompanying relocation of MS-FE. Sections S2-S7 give the implementation details.

Glioma Dice was the endpoint selected for this post hoc exploratory analysis (main-text Table 9 and Figure 7); the broader family contained 48 tests. No dated protocol or preregistration was available. Neither these hypotheses nor the interpretation boundaries are presented as prospectively fixed or confirmatory. The algorithmic and task-sharing contrasts are not ranked against the contamination contrast as like-for-like effects.

HR-MSFE, high-resolution multi-scale feature enhancement: the Section S4 module relocated to the 128-channel, 64x64 skip, as described in Section S5. HD95 is the 95th-percentile Hausdorff distance.

S10. Calibration and limits of the near-duplicate screen

The screen first removes byte-identical stored images by SHA-256. It then computes a 64-bit perceptual hash with ImageHash 4.3.1 and Pillow 10.3.0: an image is converted to 8-bit grayscale, resized to 32x32 pixels with Lanczos resampling, transformed by a two-dimensional discrete cosine transform, and encoded by comparing the upper-left 8x8 coefficients, including the direct-current coefficient, with their median. Two images are linked when they share the class label and imaging plane and their hash codes differ by at most six bits (tau=6). Connected components of this graph are inferred image-similarity groups, not verified patient identities (main-text Sections 2.7-2.8 and Algorithm 1).

Table S3 reports the number of training images directly linked to the frozen internal test set as the pHash Hamming threshold varies. At tau=6, 778 training images had a direct same-label, same-plane link. Screening removed 1,174 images (23.7% of 4,955) because removal operated on connected components of the same graph over the combined training and test sides. An image could therefore be excluded through a transitive component without a direct test-set link. All removals were from the training side; the 995-image test set was never reduced, and the screened development pool contained 3,781 images.

Table S3. Sensitivity of direct train-to-test pHash links to the Hamming threshold.

pHash Hamming threshold	Training images linked to the frozen internal test set
0 (exact pHash)	96
2	164
4	380
6 (used in this study)	778
8	1,342
10	2,094
12	2,887

These are direct image-link counts, not counts of verified patients or of all images removed through transitive components. Equal pHash values do not imply byte identity.

For base-rate context, a patient-wise split of a converted two-dimensional meningioma cohort still gave a 60.0% raw near-duplicate rate at tau=6, compared with 70.0% under a slice-wise split. The BRISC2025 distributed split gave 54.6%, the Nickparvar collection gave 61.3%, and seven BRISC test images were byte-identical to training images. These contextual rates are distinct from the direct training-image counts in Table S3. They show why a raw near-duplicate rate needs a dataset-specific base-rate comparison and do not determine how many BRISC links represent same-patient overlap.

The surrogate was calibrated on an independent public brain-tumor dataset with patient identifiers (3,064 slices from 233 patients). At tau=6, 1,470 of 1,802 direct pHash-linked pairs came from the same patient, giving pair-level precision 0.816. Those links recovered 0.058 of all same-patient slice pairs. These values describe direct links in the Cheng dataset. They do not estimate the precision of transitive connected components, and transfer to BRISC is not established. The screen is therefore a calibrated near-duplicate filter rather than patient-level separation. It cannot show that most of the 1,174 BRISC exclusions were true same-patient contamination or certify that no patient remained on both sides of the split.

Table S4. Calibration of direct pHash links against ground-truth patient identifiers (25,413 same-patient slice pairs).

Hamming threshold	pHash links	Truly same-patient links	Precision	Recall over same-patient pairs
2	177	169	0.955	0.007
4	704	648	0.920	0.025
6 (used)	1,802	1,470	0.816	0.058
8	4,024	2,471	0.614	0.097
10	9,107	3,558	0.391	0.140

Validation data: Cheng J, Huang W, Cao S, Yang R, et al. Enhanced performance of brain tumor classification via tumor region augmentation and partition. PLoS One. (2015) 10:e0140381. doi: 10.1371/journal.pone.0140381; data DOI: 10.6084/m9.figshare.1512427.

The threshold was selected from the calibration sweep in Tables S3-S4, not from BRISC test outcomes. Relaxing tau to 8 or 10 increases recall but reduces precision; tightening it to 2 or 4 further reduces recall. Because no dated preregistration exists, tau=6 is reported as a fixed analysis choice rather than a prospectively specified criterion.

Replacement-based contamination experiment

The main contamination experiment is not a comparison against training on the original unscreened 4,955-image pool. For each screened fold, a replacement-based contaminated training fold has the same size (2,997-3,061 images), exactly the same tumor-class-by-imaging-plane counts, and the same validation fold. Within each stratum, 1,135-1,151 screen-negative records per fold were replaced by detected test-linked records. Both conditions were trained with seeds 42, 1337, 2024, 3407, and 5090 and evaluated on the same frozen 995-slice test set. Per-image constituent-source provenance was unavailable, so source-dataset matching could not be implemented. Training/validation group separation is established only under the implemented detector, not as verified patient independence (main-text Section 2.7).

S11. Bottleneck attribution analysis (Figure 10 of the main text)

The classification head reads the 512-channel, 16x16 bottleneck also used by the segmentation decoder. At this selected layer, global average pooling makes the gradient for each bottleneck channel spatially constant. For the ensemble-predicted class, channel weights were the spatial means of the target-logit gradients. The weighted map was ReLU-rectified, L1-normalized within each fold, averaged equally over five folds, and bilinearly upsampled to 256x256 with align_corners=false. Models were in evaluation mode with dropout disabled and exponential-moving-average weights loaded (main-text Section 3.6).

Because the head also contains LayerNorm, GELU, dropout, and two linear layers, this attribution is not standard linear CAM. Earlier spatial encoder layers would also be possible attribution targets. Each map was scored as a per-pixel detector of the annotated lesion in 821 correctly classified masked slices. Mean pixel AUROC was 0.750 for glioma, 0.705 for pituitary, and 0.554 for meningioma. No formal chance-level test was performed, so the meningioma value is described as closer to 0.5 than the other class values, not as statistically indistinguishable from chance.

Dural attachment, peripheral location, and mask-reference mismatch are possible explanations for the meningioma pattern, not demonstrated mechanisms. Meningioma also had the lowest recall (0.904) and accounted for all 20 tumor slices classified as no tumor. Glioma pixel AUROC did not vary monotonically across lesion-area quartiles (0.722, 0.805, 0.759, and 0.714), and the intervals overlapped. This does not establish the absence of a size effect. Pixel AUROC is less mechanically constrained by lesion-area fraction than the enrichment ratio, but it is not strictly size-invariant. Bottleneck Grad-CAM remains a post hoc, coarse-resolution attribution and does not explain or causally localize the classifier's decision.

S12. Cluster bootstrap over pseudo-patient groups

Table S5 compares slice and pseudo-patient cluster resampling for the listed endpoints of the earlier frozen seed-42 analysis. This ensemble has accuracy 0.9648 and is distinct from the seed-42 ensemble in the five-seed rerun, whose accuracy is 0.9638 (main-text Section 2.6). The frozen internal test set contains 995 slices in 872 inferred groups (mean size 1.14, maximum 15; 80.4% of slices are singleton groups). The 856 masked slices form 747 inferred groups, and no inferred group spans classes. Both columns of Table S5 use the same cached predictions, seed, and 5,000 replicates; paired comparisons resample the same units for both arms.

Table S5 is a separate matched-resampling sensitivity analysis, not a replacement for the main-text confidence intervals. As specified in main-text Section 2.6, the three mean-Dice estimators in Table 1 use a 5,000-resample case bootstrap (seed 42), other Table 1 entries use a 1,000-resample slice bootstrap, and Table 8 paired calibration differences use a 2,000-resample paired case bootstrap (seed 42). The arithmetic-mean true-class Dice comparisons described in main-text Section 3.3 are separate from the Hodges-Lehmann estimates in Table 7. Their interval limits need not equal those of the 5,000-resample sensitivity analysis reported here.

For the endpoints shown in Table S5, cluster intervals were at most 13% wider than slice intervals, and the pooled mean class Dice width was unchanged after rounding (ratio 1.000). The listed exploratory interval classifications were unchanged: the multi-task glioma and whole-tumor Dice difference intervals excluded zero, whereas the meningioma and pituitary intervals included zero. Equal-width ECE differences remained statistically non-zero in this sensitivity analysis for ResNet50 and ConvNeXt-Tiny under cluster resampling (two-sided bootstrap p=0.026 and p=0.030). The SwinV2-Tiny difference remained non-significant (p=0.136). These are post hoc, non-confirmatory comparisons and do not establish patient-level calibration superiority.

The observed design effects were 1.036 for accuracy and 1.027 for whole-tumor Dice. These modest changes are conditional on the inferred grouping, whose direct-link recall was only 5.8% in Cheng. As a sensitivity scenario, replacing the observed mean group size with 13.15 slices per patient from the Cheng collection, while holding the estimated intracluster correlation fixed, gives design effects of 4.14 and 3.25. This calculation is an extrapolated scenario, not a bound, measurement, or patient-level uncertainty estimate for BRISC.

Primary hierarchical intervals for the five-seed contamination contrast

The primary contamination intervals in main-text Table 12 use a different procedure: a 10,000-resample hierarchical percentile bootstrap with random seed 20260828. Each replicate samples five training seeds with replacement, samples inferred test-set pseudo-patient clusters with replacement within each selected seed, retains all member slices with multiplicity, recomputes both conditions on the same sampled indices, and averages the paired contaminated-minus-screened differences across the sampled seeds. The 2.5th and 97.5th percentiles define the 95% interval. A Student-t interval over the five seed differences (df=4) is only an indicative small-sample summary, not the primary interval.

For HD95, the primary paired analysis uses slices with finite HD95 in both conditions within a seed (n=847, 850, 849, 849, and 851). The sensitivity analysis assigns undefined HD95 the maximum 256x256 image diagonal (362.04 pixels) and includes all 856 masked slices. The descriptive whole-tumor HD95 entry in Table S5 instead belongs to the earlier frozen ensemble and uses its finite cases (n=850). Neither the Table S5 intervals nor the hierarchical intervals establish patient-level independence.

Table S5. Slice-resample versus pseudo-patient cluster-resample intervals on the frozen internal test set.

Endpoint	Point	Slice-resample CI	Pseudo-patient cluster CI	Width ratio
Accuracy	0.9648	0.9528 to 0.9759	0.9519 to 0.9766	1.07
Macro-F1	0.9614	0.9482 to 0.9736	0.9463 to 0.9746	1.11
Macro-AUC	0.9984	0.9974 to 0.9993	0.9973 to 0.9993	1.04
ECE (15 equal-width bins)	0.0324	0.0274 to 0.0442	0.0267 to 0.0446	1.07
Brier score	0.0540	0.0385 to 0.0703	0.0372 to 0.0728	1.12
Mean class Dice (pooled)	0.7941	0.7743 to 0.8130	0.7746 to 0.8134	1.00
Mean class Dice (class macro)	0.7899	0.7703 to 0.8088	0.7707 to 0.8095	1.01
Mean class Dice (per-case foreground)	0.8127	0.7985 to 0.8267	0.7985 to 0.8274	1.02
Glioma Dice	0.7162	0.6810 to 0.7497	0.6803 to 0.7500	1.01
Meningioma Dice	0.8502	0.8241 to 0.8738	0.8236 to 0.8748	1.03
Pituitary Dice	0.8035	0.7811 to 0.8239	0.7807 to 0.8241	1.01
Whole-tumor Dice	0.8290	0.8169 to 0.8407	0.8164 to 0.8415	1.05
Whole-tumor HD95 (pixels)	8.343	7.439 to 9.317	7.372 to 9.409	1.08
Glioma Dice, multi-task minus single-task (90% CI)	+0.0279	0.0134 to 0.0433	0.0129 to 0.0440	1.04
Meningioma Dice, multi-task minus single-task (90% CI)	-0.0015	-0.0150 to 0.0121	-0.0165 to 0.0130	1.09
Pituitary Dice, multi-task minus single-task (90% CI)	+0.0066	-0.0038 to 0.0166	-0.0045 to 0.0173	1.07
Whole-tumor Dice, multi-task minus single-task (90% CI)	+0.0057	0.0014 to 0.0102	0.0006 to 0.0106	1.13
Delta ECE, unified minus pretrained ResNet50	-0.0110	-0.0217 to -0.0016	-0.0233 to -0.0014	1.09
Delta ECE, unified minus pretrained ConvNeXt-Tiny	-0.0126	-0.0230 to -0.0020	-0.0232 to -0.0014	1.04
Delta ECE, unified minus pretrained SwinV2-Tiny	-0.0058	-0.0159 to 0.0020	-0.0166 to 0.0021	1.05

Both columns use the same cached predictions, seed, and 5,000 replicates. The width ratio is cluster-interval width divided by slice-interval width. Intervals are 95% unless the row is explicitly labeled 90% CI. The 90% rows concern arithmetic-mean true-class sensitivity analyses, not Hodges-Lehmann shifts.

This table reports the earlier frozen ensemble and its matched-resampling sensitivity analysis. It does not replace the differently resampled main-text intervals or the five-seed hierarchical contamination analysis. ECE, expected calibration error; HD95, 95th-percentile Hausdorff distance.

S13. Dataset composition and near-duplicate quality control

Table S6 gives the distributed classification partition, exact-duplicate removal, screened development-pool size, and frozen internal test set. Five-fold development used the 3,781-image screened pool. The original 4,955-image pool is the exact-deduplicated, pre-screening pool, not the contaminated training condition in the main experiment. The replacement-based condition is constructed separately within each screened fold, preserving training size, tumor-class-by-plane composition, and the validation fold (main-text Sections 2.1, 2.7, and 2.8). Both contamination conditions use the same frozen 995-image test set, including 856 images with supplied segmentation masks and 139 no-tumor images.

Table S6. Dataset composition, near-duplicate quality control, and the matched contamination design.

Item	Count / status	Note
BRISC2025 original partition	5,000 / 1,000	Training side / test side as distributed.
After SHA-256 exact-duplicate removal	4,955 / 995	45 training-side and 5 test-side images removed. The 995-image test side is then frozen.
Screened development pool	3,781	Pool used for grouped five-fold training and validation; this is not the number of training images in every fold.
Frozen internal test set	995	Same test images for both contamination conditions and the internal comparisons.
Test images with supplied masks	856	Segmentation evaluation; the other 139 test images are no-tumor images.
Training images excluded by near-duplicate screening	1,174 (23.7% of 4,955)	Excluded through detected test-linked components. These are image counts, not verified patient counts.
Training size per screened or matched contaminated fold	2,997-3,061	Each contaminated fold exactly matches the size and tumor-class-by-plane counts of its screened counterpart.
Within-stratum replacements per contaminated fold	1,135-1,151	Screen-negative training records replaced by detected test-linked records; validation folds unchanged.
Training seeds in the contamination experiment	42, 1337, 2024, 3407, 5090	Five seeds for each condition; test-slice identifiers are unchanged.
Inferred test-set groups	872 across 995 slices; 747 across 856 masked slices	pHash-connected components, not verified patient identifiers.

Constituent source-dataset provenance is unavailable at image level, so source-matched replacement is not established. The pHash surrogate has low recall for known same-patient pairs; screening and grouped resampling do not certify patient-exclusive partitions. Performance estimates remain exploratory slice-level benchmark estimates.
